# NLRP3 Inflammasome and Extracellular Vesicles in Knee Osteoarthritis: Molecular Mechanisms, Biomarkers, and Translational Therapeutic Strategies

**DOI:** 10.3390/biology15211959

**Published:** 2026-11-01

**Authors:** Jing He, Jing Cai, Xinyu Shi, Jiaqi Chen, Da Liu, Nan Zhang, Ye Jin

**Affiliations:** 1 School of Pharmacy, Changchun University of Chinese Medicine, Changchun 130117, China; hejing1618@ccucm.edu.cn (J.H.); 24204702023@stu.ccucm.edu.cn (J.C.); liuda_1986@163.com (D.L.); 2 College of Traditional Chinese Medicine, Changchun University of Chinese Medicine, Changchun 130117, China; caijing@ccucm.edu.cn; 3 Jilin Ginseng Academy, Changchun University of Chinese Medicine, Changchun 130117, China; shixy4242@ccucm.edu.cn; 4 Public Laboratory Centre, Changchun University of Chinese Medicine, Changchun 130117, China; 5 Northeast Asian Institute of Traditional Chinese Medicine, Changchun University of Chinese Medicine, Changchun 130117, China

**Keywords:** NLRP3 inflammasome, osteoarthritis, extracellular vesicles, targeted therapy

## Abstract

Background/Objectives: Osteoarthritis (OA) is the most prevalent degenerative joint disease worldwide, characterized by progressive cartilage degeneration, chronic synovial inflammation, and abnormal subchondral bone remodeling, with high disability rates and a substantial disease burden. The knee joint is the most commonly affected site in OA; therefore, this review focuses on knee OA. Traditionally, knee OA has been regarded as cartilage degeneration caused solely by mechanical wear; however, accumulating preclinical and translational evidence supports NLR family pyrin domain-containing protein 3 (NLRP3) inflammasome activation as an important contributing factor in specific inflammatory and metabolic knee OA phenotypes. This review aims to integrate the potential roles of the NLRP3 inflammasome and extracellular vesicles (EVs) in knee OA, and to explore their exploratory biomarker value and investigational therapeutic potential. Methods: This narrative review synthesizes the current published knowledge regarding NLRP3 inflammasome biology, knee OA pathogenesis, and translational research on extracellular-vesicle-based diagnostics and therapeutics. The relevant peer-reviewed literature was identified via literature searches in major biomedical databases up to September 2026. The authors assembled references based on thematic fit to the review scope, study quality, and maturity of available evidence. Original reports, clinical trials, meta-analyses, published systematic reviews, and clinical guidelines were considered; the non-peer-reviewed grey literature was not used as primary supporting evidence. Where conflicting study outcomes exist in the published literature, human clinical data and randomized controlled trial evidence were given greater interpretive weight relative to preclinical in vitro and animal-model findings. Results: This review proposes that the coupling among inflammatory responses, pyroptosis, and EVs-mediated signal transmission may constitute an important pathological link in knee OA progression. NLRP3 integrates mechanical, metabolic, and aging signals in chondrocytes, synovial macrophages, and subchondral bone cells, triggering pyroptosis and matrix degradation; it also mediates transcellular and trans-tissue cascade amplification of inflammatory signals via EVs and may drive the progression from focal injury to pan-articular degeneration. Small-molecule inhibitors targeting NLRP3 have evolved from the early experimental probe MCC950 to a new generation of tissue-selective clinical candidates; however, no NLRP3 inhibitor has yet entered dedicated clinical trials for knee OA. NLRP3-pathway molecules associated with EVs have exploratory potential as liquid biopsy biomarkers reflecting local joint inflammatory burden and may be investigated for early diagnosis and patient stratification in knee OA; engineered EVs may serve as targeted delivery vehicles, and preclinical studies have demonstrated inflammatory-microenvironment-responsive NLRP3 inhibition and cartilage-specific enrichment. Conclusions: NLRP3-targeted intervention is an investigational strategy with potential disease-modifying intent in knee OA, but it remains at the preclinical and early translational stages. To date, no clinical evidence demonstrates that selective NLRP3 inhibition improves pain, function, structural progression, or other patient-centered outcomes in knee OA; therefore, disease modification by NLRP3 inhibition remains unproven. Its early translational research faces core bottlenecks, including long-term local medication safety, intra-articular delivery efficiency, the high heterogeneity of knee OA, and standardized quality control of vesicle products. Future efforts should focus on vesicle-based multi-omics inflammatory subtyping, engineered vesicle systems that combine deep cartilage penetration with long-term joint retention, and multi-mechanism combination therapies. These approaches may generate hypotheses for future research into whether knee OA treatment can eventually move beyond symptomatic relief toward disease modification; however, this transition remains unproven and requires dedicated clinical trials with appropriate clinical endpoints. It should also be acknowledged that the available evidence is not entirely consistent: some studies have failed to demonstrate that stress-induced cartilage degradation is NLRP3-dependent, underscoring the importance of OA heterogeneity. Current evidence is largely derived from preclinical studies, and validation in human studies remains limited. It should be emphasized that, although NLRP3 inhibitors have reached clinical stages in other indications (such as neurodegenerative diseases, metabolic diseases, and cardiovascular diseases), these results primarily validate the druggability of the NLRP3 target itself and cannot be directly equated with clinical efficacy in knee OA. Dedicated clinical trials specifically designed for knee OA populations with appropriate clinical endpoints are required to establish therapeutic benefit.

## Simple Summary

Knee Osteoarthritis (knee OA) is a common degenerative joint disease, traditionally attributed to mechanical wear. Recent studies have identified the NLRP3 inflammasome as an important mediator of chronic joint inflammation and tissue destruction in specific inflammatory and metabolic OA phenotypes. This review integrates the mechanisms of NLRP3-induced chondrocyte pyroptosis and extracellular vesicle-mediated inflammation dissemination, reviews the small-molecule inhibitors targeting this pathway and vesicle-based diagnostic and therapeutic strategies, and discusses their prospects and translational challenges for early diagnosis and endotype-directed investigational approach.

## 1. Introduction

Knee osteoarthritis (knee OA) is the most prevalent degenerative joint disease worldwide. It is characterized by progressive cartilage degeneration, chronic synovial inflammation, and abnormal subchondral bone remodeling, and is associated with high disability rates and a substantial disease burden. For a long time, knee OA has been regarded as a mechanically stress-driven degenerative disorder, and clinical management has primarily relied on symptomatic relief, such as nonsteroidal anti-inflammatory drugs (NSAIDs) and intra-articular sodium hyaluronate injections, and no disease-modifying drug has yet been shown to effectively slow disease progression in knee OA [1,2,3]. The knee joint is the most commonly affected site in OA. It possesses an intact synovium–cartilage–subchondral bone structure and is the most extensively studied model for mechanistic research and local intervention studies; therefore, this review focuses on knee OA.

The innate immune system serves as the first line of host defense against exogenous pathogens and endogenous injury, and its precise sensing capacity and appropriately calibrated response intensity are fundamental to maintaining tissue homeostasis and immune balance. Inflammasomes are multiprotein complexes assembled in the cytosol and are among the important regulatory modules involved in the inflammatory network of knee OA [4].

Among them, the NLRP3 inflammasome has attracted considerable attention owing to its broad pathological relevance and well-characterized functions, and its aberrant activation has been implicated in multiple chronic inflammatory diseases. NLRP3 activation follows the canonical “two-signal” model and ultimately mediates the maturation and release of IL-1β and IL-18, and cleaves Gasdermin D (GSDMD)-mediated pyroptosis [5,6,7,8]. Detailed molecular mechanisms are described in Section 2.

Recent studies have shown that chronic low-grade inflammation is an important pathological mechanism in the development and progression of knee OA. The innate immune response, driven by damage-associated molecular patterns (DAMPs) together with multi-tissue crosstalk among cartilage, synovium, and subchondral bone, contributes to joint tissue destruction. The NLRP3 inflammasome can integrate multiple danger signals related to mechanical stress, metabolic disturbance, and aging, and serves as a key regulatory node in the inflammatory network of knee OA [9,10,11]. Extracellular vesicles (EVs), in turn, can mediate the dissemination of inflammatory signals among different cells within the joint, thereby amplifying local injury responses [12,13]. Notably, knee OA is highly heterogeneous, and NLRP3 hyperactivation appears to play a more prominent role in inflammation- and metabolism-related subtypes. However, it should be acknowledged that not all preclinical and translational studies support a central and universal role for NLRP3 in OA pathogenesis; some studies have reported that stress-induced cartilage degradation can proceed through NLRP3-independent pathways, and NLRP3 targeting has not consistently demonstrated efficacy across different experimental models, highlighting the importance of considering disease heterogeneity and conflicting evidence.

The above evidence suggests that targeting NLRP3 is a candidate investigational strategy that may have disease-modifying potential in selected knee OA subtypes; however, this has not been established in humans. Challenges such as low local drug delivery efficiency and unclear long-term safety remain major obstacles to clinical implementation. In recent years, the development of NLRP3 inhibitors has progressed rapidly, evolving from the early probe MCC950 to a new generation of tissue-selective candidates with several candidates entering Phase I/II clinical trials for indications other than knee OA. Nevertheless, most clinical development has focused on non-OA indications, and clinical evidence specific to knee OA remains lacking [14,15,16]. In this review, a rigorous distinction is maintained between evidence directly derived from knee OA models and human OA specimens, and evidence extrapolated from NLRP3 inhibitor studies in other indications; while the latter are valuable for establishing target validation and proof of concept, they do not constitute evidence for knee OA efficacy and should be interpreted with caution.

**Figure 1:**
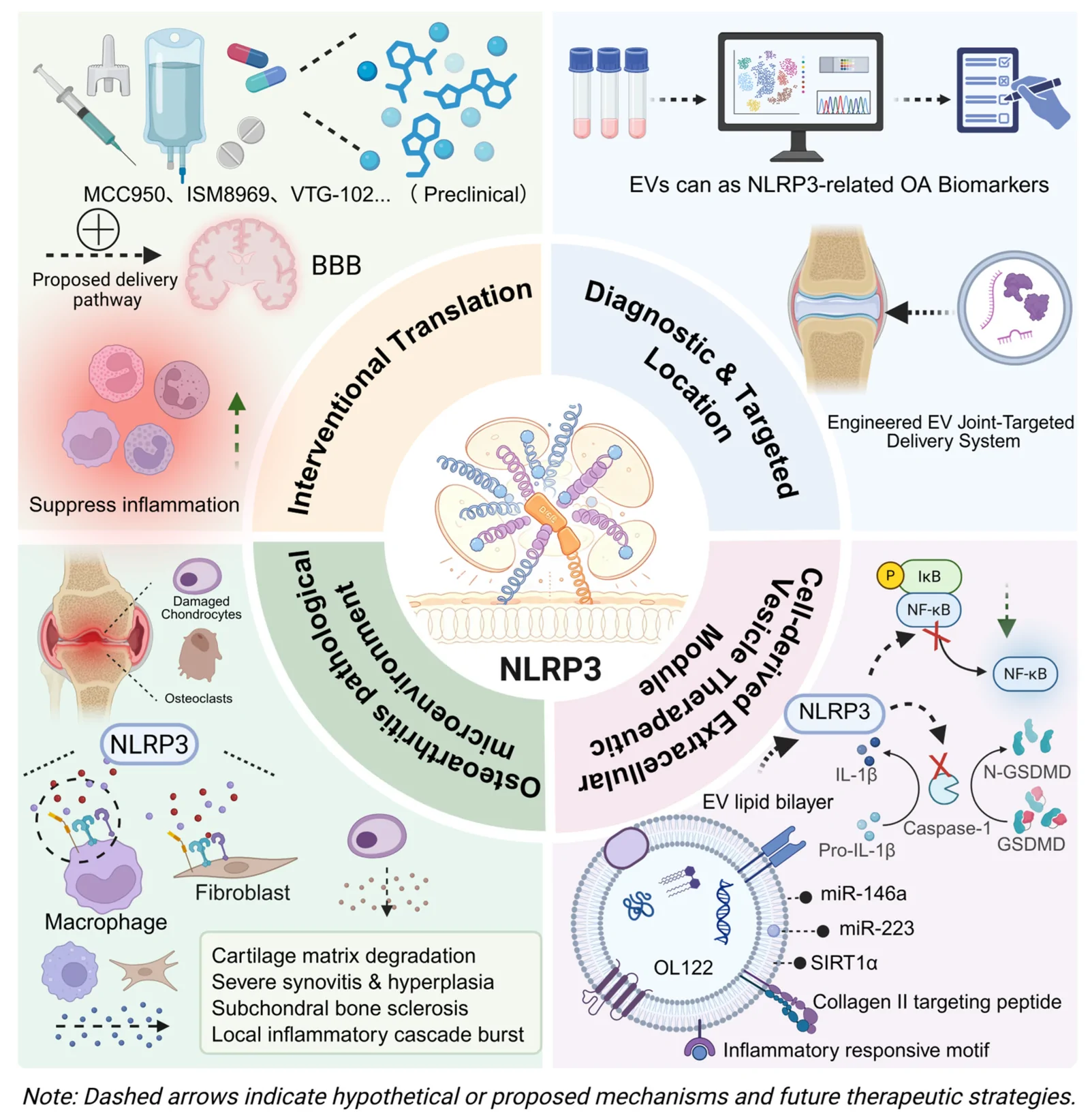
NLRP3 inflammasome: proposed pathological network and investigational translational strategies in knee OA. This schematic presents the proposed pathological regulatory network of the NLRP3 inflammasome in knee OA and its translational applications. The left panel depicts the potential of EVs as biomarkers: synovial fluid-derived EVs can carry inflammatory molecules such as NLRP3, active caspase-1, and IL-1β, while circulating EVs may allow detection of chondrocyte-specific type II collagen and NLRP3; in combination with non-coding RNAs including miR-146a and miR-223, these EV-based markers are being explored to reflect articular inflammatory burden, stratify degenerative stages, and aid early diagnosis and patient stratification in knee OA. The right panel illustrates engineered EV-based joint-targeted delivery systems: through surface modification with type II collagen-binding peptides and incorporation of inflammation-responsive elements, these engineered EVs may achieve targeted enrichment at injured joints and suppress NLRP3 inflammasome activation across multiple cell types, including chondrocytes, synovial macrophages, and osteoclasts, thereby potentially attenuating pathological phenotypes such as cartilage matrix degradation, synovial inflammatory hyperplasia, and subchondral bone sclerosis. The left panel summarizes exploratory biomarker evidence, whereas the right panel depicts mainly preclinical therapeutic strategies; solid arrows indicate mechanisms supported by experimental evidence, and dashed arrows indicate proposed or hypothetical mechanisms.

EVs, as emerging mediators of intercellular communication, offer a new dimension for investigating NLRP3-targeted intervention and theranostic strategies in knee OA, owing to their natural biocompatibility, low immunogenicity, and molecular delivery capabilities. EVs derived from chondrocytes and synovial macrophages in the synovial fluid and serum of patients with knee OA can carry NLRP3 pathway molecules, initially showing potential as biomarkers of disease progression [12,13]. EVs derived from mesenchymal stem cells (MSCs) and other sources can suppress NLRP3 activation by delivering functional non-coding RNAs such as miR-146a and miR-223; engineered EVs, in turn, show potential as delivery vehicles for NLRP3 inhibitors and small interfering RNA (siRNA) [17,18,19]. However, the precise molecular mechanisms by which EVs regulate NLRP3 have not been fully elucidated, and the absence of standardized large-scale preparation, quality control, and in vivo safety evaluation systems also limits their early translational research (Figure 1).

Unlike previous reviews that mainly summarized NLRP3 inflammasome biology, MSCs-derived EV therapies, or EV-based biomarkers as separate topics, this narrative review proposes an integrated framework linking NLRP3 activation with EV-mediated inflammatory dissemination, with a focus on knee OA. Specifically, we discuss how mechanical overload, metabolic stress, and aging-related signals converge on NLRP3 activation in chondrocytes, synovial macrophages, and subchondral bone cells, and how EVs carrying NLRP3, active caspase-1, and IL-1β amplify this inflammatory cascade across joint tissues. This integrated perspective may help explain the phenotypic heterogeneity of knee OA—particularly the greater prominence of NLRP3-driven pathology in inflammatory and metabolic subtypes—and provide a basis for investigating stratified interventions targeting the NLRP3-EV axis, maintaining a rigorous distinction between preclinical evidence and clinical translation, and developing EV-based patient stratification and investigational therapeutic strategies. Against this background, this review focuses on knee OA and systematically reviews the molecular regulatory mechanisms of the NLRP3 inflammasome, its pathological roles in different joint tissues, advances in targeted inhibitor development, and the translational potential of EVs in NLRP3-related diagnosis and therapy. It also discusses the core bottlenecks and future directions of the current early translational research.

## 2. Advances in Molecular Mechanisms and Pathophysiological Functions of the NLRP3 Inflammasome

Unless otherwise specified, the molecular mechanisms summarized in this section are derived mainly from non-OA cell and animal models; direct evidence in human OA cartilage or synovium remains limited. The pathological progression of knee OA involves multiple signals. Among them, the NLRP3 inflammasome, owing to its broad capacity to sense danger signals, is regarded as one of the molecules potentially involved in integrating these pathological stimuli. However, NLRP3 activation is not a simple linear process, but is jointly influenced by multiple layers of mechanisms, including transcriptional regulation, post-translational modifications (PTMs), subcellular localization, and organellar damage. Therefore, this chapter will review the regulatory network of the NLRP3 inflammasome from two core perspectives—molecular assembly and signal transduction—and further elaborate on its potential pathophysiological roles in different cells in knee OA, thereby providing a basis for the subsequent discussion of targeted intervention strategies.

### 2.1. Molecular Regulatory Network of NLRP3 Inflammasome Activation

The NLRP3 inflammasome is an evolutionarily conserved multiprotein signaling platform in the innate immune system [20], functioning as both a “danger signal sensor” and an “inflammatory effector amplifier” [21], and playing indispensable roles in host defense, tissue homeostasis maintenance, and injury repair [22,23,24]. Unlike canonical pattern-recognition receptors, NLRP3 exhibits no strict ligand-binding specificity. Its activation does not rely on direct recognition of a single ligand; instead, it initiates assembly by integrating diverse danger signals that trigger intracellular homeostatic perturbation [25], including ionic imbalances, organellar membrane damage, oxidative stress, and metabolite accumulation [26]. Its functional execution is highly dependent on precise conformational regulation, post-translational modification networks, and subcellular compartmentalization [27,28,29]. In recent years, techniques such as cryo-electron microscopy (cryo-EM) structural determination [11], super-resolution microscopy [30], and liquid–liquid phase separation (LLPS) [31,32] have advanced the structural elucidation of NLRP3, providing actionable sites for targeted intervention. In knee OA, these mechanisms may be associated with aberrant activation of chondrocytes and synovial macrophages.

#### 2.1.1. Structural Basis and Assembly Dynamics

The NLRP3 protein comprises three domains: PYD, NACHT, and LRR [33], which mediate downstream recruitment [34]; oligomerization [35,36,37], and signal sensing/autoinhibition [38], respectively. In the resting state, LRR folds back onto NACHT to prevent spontaneous aggregation [35,39,40,41]. NEK7 acts as a licensing factor promoting NLRP3 activation, and NLRP3-NEK7 co-assemble into disc-shaped oligomers that provide a nucleation interface for apoptosis-associated speck-like protein containing a CARD (ASC) [42].

#### 2.1.2. Integration Mechanisms of Upstream Activation Signals

It is now widely accepted that NLRP3 activation follows a two-step model, namely the priming and activation phases [43,44,45]. Recent studies have progressively revealed that this process is not a simple linear signaling cascade, but rather an integrated regulatory network involving multiple signals and organelles [46,47].

##### Priming Phase: Transcriptional Regulation and Post-Translational Modifications

The core function of the priming phase is to increase NLRP3 protein abundance and lower its activation threshold, a process primarily mediated by Toll-like receptor (TLR) ligands, TNF-α, and other pro-inflammatory signals through NF-κB pathway activation [48,49]. In knee OA, mechanical stress, cartilage matrix degradation fragments, and DAMPs can activate NLRP3 transcription in chondrocytes and synovial macrophages via the TLR4/NF-κB pathway [50]. In the classical view, NF-κB nuclear translocation directly upregulates the transcription of NLRP3, pro-IL-1β, and pro-IL-18, increasing basal protein expression [51,52]; subsequent studies have confirmed that the regulation of post-translational modifications is a critical determinant of the activation threshold [53]. In knee OA, persistent activation of the NF-κB pathway is associated with elevated NLRP3 expression in chondrocytes and synovial macrophages, but the specific transcriptional regulatory mechanisms still require further investigation.

Ubiquitination is involved in regulating NLRP3 activation, and deubiquitinating enzymes can remove inhibitory ubiquitin chains from NLRP3, thereby promoting its activation [54]. In knee OA, the regulatory role of deubiquitinating enzymes on NLRP3 remains to be validated; current evidence is mainly derived from other cell models. Phosphorylation, SUMOylation, acetylation, and other modifications jointly regulate the activation threshold of NLRP3. In knee OA, mechanisms such as PKA/AKT-mediated inhibitory phosphorylation of NLRP3 and SENP6-mediated de-SUMOylation have been preliminarily studied in chondrocytes or synovial cells, but their specific roles still require validation [55,56,57,58].

##### Activation Signals: Convergence of Ion Imbalance, Organellar Damage, and Metabolic Stress

The activation phase is the critical step in which NLRP3 undergoes conformational transition and completes oligomeric assembly, with multiple upstream signals ultimately converging on common cellular events [59]. Among these, reduced intracellular potassium concentration is currently the most well-established common upstream event, and the activation effects of most NLRP3 agonists depend on a decrease in intracellular potassium concentration [60]; calcium signaling disturbance and hydrogen ion leakage caused by lysosomal rupture can synergistically promote activation [61,62,63,64]. In knee OA, mechanical stress, adenosine triphosphate (ATP)-P2X7 signaling, and calcium signaling disturbance may trigger potassium efflux, and elevated synovial ATP levels may participate in this process. Organellar membrane damage is the subcellular platform for signal convergence [65]: Golgi membrane remodeling generates dispersed trans-Golgi network (dTGN) vesicles carrying phosphatidylinositol-4-phosphate (PtdIns4P), and NLRP3 can directly bind PtdIns4P through its conserved polybasic region, completing recruitment and initial oligomerization on the dTGN surface [66]; lysosomal membrane permeabilization releases cathepsin B, which cleaves NLRP3 or its upstream regulatory molecules [62,63,67]; mitochondrial damage releases mitochondrial antiviral signaling protein (MAVS) and oxidized mitochondrial DNA (ox-mtDNA), which directly bind and activate NLRP3 [51,52,53,54,55]. In knee OA, cartilage matrix degradation fragments, calcium pyrophosphate crystals, and uric acid crystals can trigger lysosomal damage, while mechanical overload and metabolic disturbance can lead to mitochondrial ROS and ox-mtDNA release, participating in NLRP3 activation in chondrocytes and synovial macrophages.

Oxidative stress and metabolic disturbance synergistically amplify activation signals. Reactive oxygen species (ROS) exert synergistic effects through oxidative modification of NLRP3 or its regulatory proteins. In the classical TXNIP pathway, under oxidative stress, thioredoxin dissociates from TXNIP, and free TXNIP binds to and activates NLRP3 [68,69]. Metabolic signals such as free fatty acids under hyperlipidemia and advanced glycation end products (AGEs) can indirectly activate NLRP3 through oxidative stress and organellar damage [70,71,72]. In metabolic OA, obesity and lipotoxicity may activate NLRP3 through the TXNIP pathway and mitochondrial ROS. In non-canonical pathways, intracellular lipopolysaccharide (LPS) can activate caspase-4/5/11 and indirectly activate NLRP3, but direct evidence in knee OA is limited.

#### 2.1.3. Multiple Outputs of Downstream Effector Pathways

The assembled NLRP3 inflammasome recruits the adaptor ASC via PYD–PYD homotypic interactions, which in turn recruits pro-caspase-1 through CARD–CARD interactions, inducing autoproteolytic cleavage to generate active caspase-1 tetramers that initiate multiple downstream effector pathways [73,74,75,76].

Active caspase-1 specifically cleaves pro-IL-1β and pro-IL-18 to generate biologically active mature cytokines, mediating local immune cell recruitment, activation, and systemic inflammatory responses. GSDMD, releasing its N-terminal fragment, which polymerizes on the inner leaflet of the plasma membrane to form pores, leading to pyroptosis [77,78,79].

Recent studies have revealed that a sub-lethal pyroptotic state is more common in chronic inflammatory pathologies. Following GSDMD pore formation, cells can survive through ESCRT-mediated membrane repair, releasing only mature cytokines and DAMPs through membrane pores, thereby sustaining chronic low-grade inflammation [80,81]. This non-lethal activation mode represents an important basis for the persistent pathological role of NLRP3 in OA.

Furthermore, intercellular transmission of inflammatory signals further amplifies the pathological impact of NLRP3. ASC aggregates, active caspase-1, and some NLRP3 components formed in activated cells can be transmitted via EVs; the roles of EVs in NLRP3 signal transmission and therapy are detailed in Section 5.

**Figure 2:**
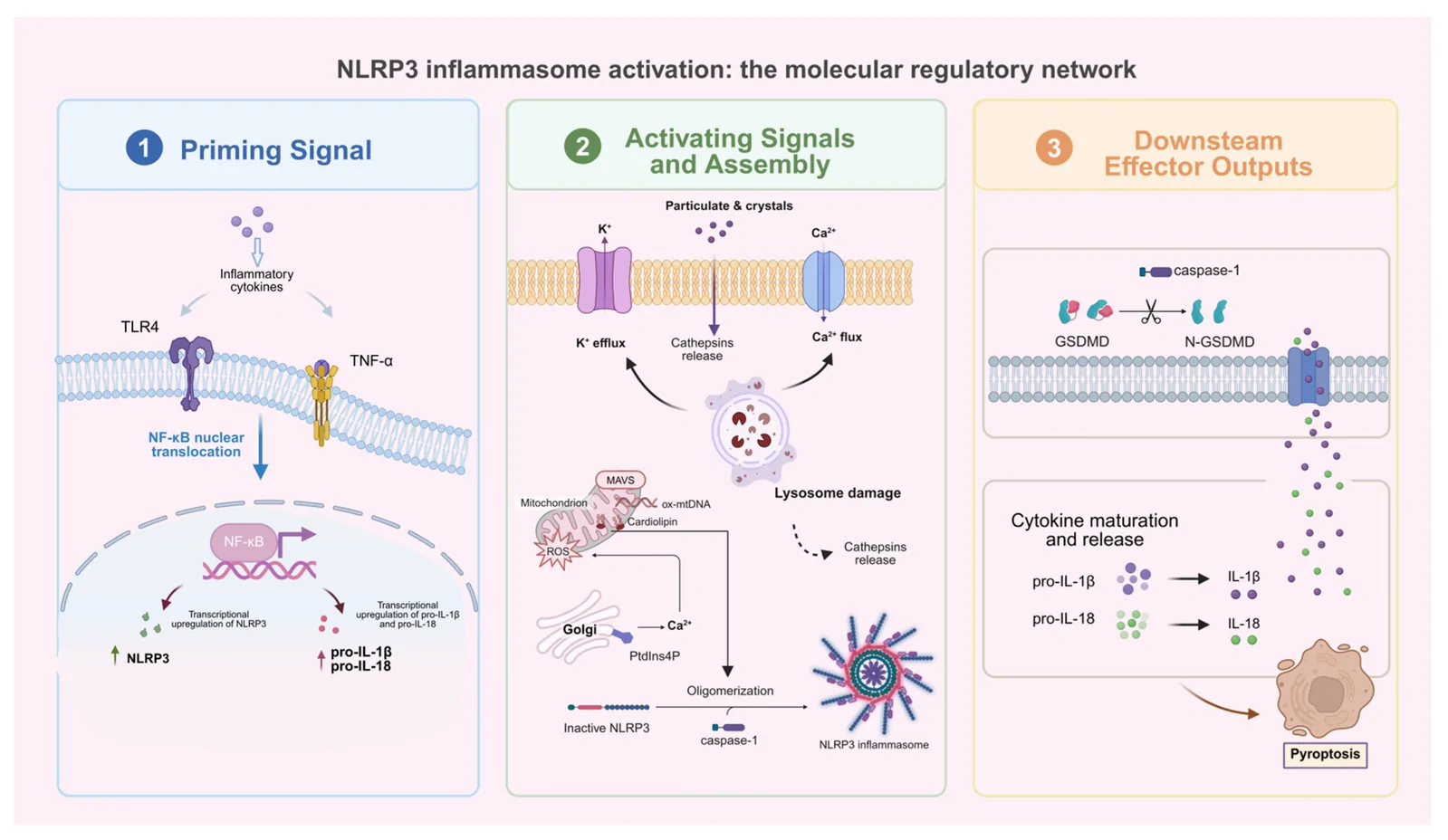
Multilayered molecular regulatory network of NLRP3 inflammasome activation. This figure illustrates the multilayered molecular regulatory network governing NLRP3 inflammasome activation. During the priming phase, pattern recognition receptors such as TLR4 sense exogenous or endogenous danger signals and upregulate the transcriptional expression of NLRP3, pro-IL-1β, and other components via the NF-κB pathway. During the activation phase, diverse upstream signals—including particulate crystals, ionic imbalances, lysosomal damage, mitochondrial dysfunction, and Golgi remodeling—converge on common cellular events such as potassium efflux, cathepsin release, and mitochondrial reactive oxygen species (mtROS) accumulation, which induce conformational changes and oligomerization of NLRP3. In the downstream effector phase, the assembled inflammasome activates caspase-1, which mediates the maturation and release of pro-inflammatory cytokines IL-1β and IL-18, and triggers pyroptosis through GSDMD cleavage, ultimately leading to tissue injury and inflammatory dissemination. The diagram also highlights the regulatory roles of post-translational modifications, including phosphorylation and deubiquitination, in modulating NLRP3 activation. Solid arrows indicate mechanisms supported by experimental evidence; dashed arrows indicate proposed or context-dependent mechanisms.

In summary, NLRP3 inflammasome activation is governed by multilayered regulation, including post-translational modifications that set the activation threshold, convergence of Golgi, lysosomal, and mitochondrial damage signals, and downstream GSDMD-mediated pyroptosis and intercellular propagation of ASC specks. These mechanisms exhibit significant heterogeneity across different tissues. In joint tissues, mechanical stress, matrix degradation fragments, and the senescence-associated secretory phenotype (SASP) collectively drive chronic NLRP3 activation in chondrocytes, synovial macrophages, and subchondral bone cells. This tissue specificity constitutes an important basis for understanding the pathological role of NLRP3 in knee OA (Figure 2).

### 2.2. Pathophysiological Roles of the NLRP3 Inflammasome in Knee OA

Under physiological conditions, NLRP3 inflammasome activation is transient and tightly regulated, serving as a necessary mechanism for pathogen clearance and tissue repair. When stimuli persist or regulatory networks become imbalanced, excessive or sustained NLRP3 activation drives chronic low-grade inflammation, and contributes to the development and progression of degenerative joint diseases [53,61,82,83,84]. The core pathological effect of NLRP3 lies in amplifying inflammatory responses through positive feedback loops, converting transient cellular stress into sustained tissue damage. Across different diseases, the triggers, the cell types expressing NLRP3, and downstream pathological effects of NLRP3 exhibit marked tissue heterogeneity; this heterogeneity is a central issue that must be addressed in early translational research and an important basis for achieving an endotype-directed investigational approach [85,86,87]. Key molecular regulatory mechanisms of NLRP3 inflammasome activation are summarized in Table 1.

In summary, a broad consensus exists on the pathogenic roles of the NLRP3 inflammasome in multisystem diseases, with a shared pathological logic: persistently present endogenous danger signals drive chronic low-grade NLRP3 activation, which amplifies inflammatory responses through positive feedback loops, ultimately leading to progressive tissue damage. At the same time, it should be recognized that the triggers, regulatory networks, and pathological outcomes of NLRP3 activation exhibit significant heterogeneity across different diseases and cell types: targeted intervention in the central nervous system (CNS) requires crossing the blood–brain barrier (BBB); treatment of joint diseases needs to achieve long-term local retention; and metabolic diseases must balance safety in the liver and immune cells.

This heterogeneity dictates that broad-spectrum systemic administration strategies can hardly achieve both efficacy and safety simultaneously, which is one of the fundamental reasons for the bottlenecks encountered in early clinical development of NLRP3 inhibitors. Future development of targeted therapies may need to advance toward highly selective molecules, tissue-specific delivery, and stratified/endotype-directed patient selection; however, these approaches remain investigational and have not been clinically validated in knee OA; novel carrier technologies such as EVs offer a new technological path for overcoming the challenges of tissue targeting and local delivery.

### 2.3. Mechanisms of OA Pain

Pain is the most prominent clinical symptom of OA, severely affecting patients’ quality of life and functional status. For a long time, OA pain was considered to arise primarily from degenerative changes in joint structures, including cartilage degeneration, abnormal subchondral bone remodeling, and osteophyte formation. However, increasing clinical evidence indicates that OA pain does not always parallel the degree of structural damage revealed by imaging, suggesting that the occurrence of pain involves multiple mechanisms.

From a pathophysiological perspective, the generation of OA pain can be divided into the following categories. First, structural progression-related pain: cartilage matrix degradation leads to irregularity of the articular surface, and abnormal mechanical loading directly stimulates intra-articular mechanoreceptors; subchondral bone microfractures and bone marrow lesions can activate intraosseous nerve endings, producing deep dull pain [106]. Second, synovitis-related pain: hyperemia, edema, and inflammatory cell infiltration in synovial tissue lead to the release of large amounts of pain mediators, which stimulate the abundant sensory innervation of the synovium [107]. Third, inflammatory mediator-mediated nociceptor sensitization: cytokines such as IL-1β and IL-18 released after NLRP3 inflammasome activation, as well as inflammatory mediators such as TNF-α, PGE2, bradykinin, and prostaglandins, can directly lower the activation threshold of intra-articular nociceptors and enhance their responsiveness to mechanical and chemical stimuli [108]. Fourth, remodeling of peripheral nociceptive pathways: chronic inflammatory stimulation can lead to proliferation of intra-articular sensory nerve fibers and increased release of neuropeptides (such as substance P and calcitonin gene-related peptide [CGRP]), establishing a state of peripheral sensitization in which even mild stimuli can elicit pain [109]. Fifth, central sensitization: persistent peripheral nociceptive input can increase the excitability of spinal dorsal horn neurons and impair central inhibitory pathways, manifesting as expansion of pain extent, reduced pain threshold, and allodynia; even when local joint lesions are controlled, pain may persist [108].

Among the above pain mechanisms, the NLRP3 inflammasome may play multiple roles. On the one hand, activation of NLRP3 in chondrocytes, synovial macrophages, and subchondral bone cells may promote the release of algogenic factors such as IL-1β, participating in the amplification of peripheral pain signals; on the other hand, NLRP3 activation products may spread among different joint tissues via pathways such as EVs, forming a pain regulatory network spanning cartilage–synovium–bone. In addition, some studies suggest that NLRP3 may also be expressed in sensory neurons or their paracrine cells, implying that it may directly participate in the generation and modulation of pain signals; however, this hypothesis still requires further validation [110].

It should be particularly noted that targeting NLRP3 to inhibit cartilage degeneration does not necessarily translate into meaningful pain relief. NLRP3 inhibition may delay structural progression, but if pain is already maintained by independent mechanisms such as central sensitization or neural remodeling, structural protection may not lead to symptom improvement. This distinction is crucial for the design of clinical therapeutic strategies: for OA patients with predominantly inflammation-mediated peripheral pain, NLRP3-targeted therapy may yield greater benefit, whereas for subtypes dominated by central sensitization or neuropathic pain, other analgesic strategies should be combined or substituted. Therefore, future endotype-directed investigational approach of OA should incorporate pain phenotype stratification, combining peripheral inflammatory biomarkers with central imaging features, to achieve cause-directed analgesia rather than generalized symptomatic analgesia.

In summary, aberrant NLRP3 activation is involved in multiple chronic inflammatory diseases, but its triggers, regulatory networks, and pathological effects exhibit marked tissue heterogeneity. In knee OA, mechanical stress, matrix degradation fragments, and the SASP collectively drive chronic NLRP3 activation in chondrocytes, synovial macrophages, and subchondral bone cells; current evidence is mainly derived from preclinical studies, and human causal evidence remains limited. Future efforts should focus on developing highly selective inhibitors, intra-articular local delivery, and biomarker-based patient stratification strategies, and novel carriers such as EVs may provide new ideas for this purpose.

## 3. Design Strategies and Progress of NLRP3 Inhibitors

The multilayered regulatory network described in Section 2 suggests that NLRP3 inflammasome activation is jointly influenced by multiple factors, including conformational changes, post-translational modifications, and subcellular localization, which also implies that targeting this pathway may face substantial druggability challenges. The NLRP3 protein has a complex structure and conformational plasticity, and the development of its inhibitors must simultaneously consider target selectivity, tissue penetration, and long-term safety. Since the advent of MCC950, the development of NLRP3-targeted drugs has undergone multiple stages, from phenotypic screening to structure-guided design, and further to AI-assisted rational design; however, most clinical development has focused on non-OA indications, and clinical evidence specific to knee OA remains lacking. Therefore, this chapter will review the design strategies, druggability optimization, and discuss their exploratory translational implications and challenges in the context of knee OA. In this chapter, we explicitly distinguish between two categories of evidence: (1) direct preclinical and clinical evidence derived from OA-specific models and specimens; (2) evidence derived from NLRP3 inhibitors initially developed for neurological, metabolic, cardiovascular, and other inflammatory diseases. Evidence from the latter confirm the druggability of NLRP3 as a target but should not be conflated with efficacy evidence for knee OA.

### 3.1. Inhibitor Classification and Mechanisms 

Based on differences in mechanism of action, chemical scaffolds, and binding sites, NLRP3 inhibitors can be classified into multiple categories. With ongoing advances in structural biology, the molecular details of inhibitor–NLRP3 interactions are being progressively elucidated, providing a solid structural foundation for rational drug design.

#### 3.1.1. Allosteric Inhibitors

Allosteric inhibitors do not directly compete for the ATP-binding site; instead, they exert inhibitory effects by stabilizing the inactive conformation of NLRP3. MCC950 (CRID3) is the first widely recognized direct NLRP3 inhibitor, possessing high selectivity and being capable of efficiently and specifically inhibiting the assembly and activation of the NLRP3 inflammasome without significantly inhibiting other inflammasomes such as AIM2, NLRC4, and NLRP1. Its mechanism of action has been progressively elucidated through structural biology: early studies suggested that MCC950 targets the NACHT domain of NLRP3, inhibiting inflammasome assembly by blocking ATP hydrolysis [111]; Hochheiser et al. (2022) resolved the high-resolution cryo-EM structure of the NLRP3–MCC950 complex, revealing its binding mode and inhibitory mechanism [112]. However, MCC950 was discontinued owing to hepatotoxicity signals observed in preclinical and early clinical studies [113,114,115,116]; nevertheless, its hepatotoxicity does not represent the toxicity profile of all NLRP3 inhibitors, and structurally distinct inhibitors require individual evaluation. Despite this, the discovery of this compound pharmacologically validated the druggability of NLRP3 as a target for further research, providing an important molecular template for the subsequent structural optimization of inhibitors. MCC950 has been shown to exert chondroprotective effects via intra-articular injection in animal models of knee OA; these findings are preclinical and do not establish clinical efficacy or disease modification in human knee OA [117].

#### 3.1.2. Covalent Inhibitors

Covalent inhibitors form irreversible covalent bonds with cysteine residues of NLRP3 through electrophilic groups, thereby achieving sustained target inhibition. Oridonin, which blocks NLRP3–NEK7 interaction via covalent modification of Cys279, is the most extensively studied natural product representative of this class [20,118]; dehydrocostus lactone selectively inhibits NLRP3 activation at extremely low concentrations [119]. Among synthetic compounds, grandiflorenic acid derivative compound 5 covalently binds to the ATP-binding site with high activity [120]. The covalent strategy offers a new direction for long-acting NLRP3-targeted drugs, although its off-target risks require careful evaluation.

#### 3.1.3. AI-Powered Rational Design: From Chemistry42 to ISM8969

Artificial intelligence (AI) is profoundly transforming the discovery paradigm for NLRP3 inhibitors. Insilico Medicine’s Chemistry42 platform integrates generative AI algorithms with multiple drug design modules, successfully advancing ISM8969 to clinical development, where it was designated as a preclinical candidate in 2024, receiving FDA IND approval in 2026, and completing first-in-human dosing in Phase I [121]. The Chemistry42 platform has also developed the LEGION tool, generating a large-scale dataset of approximately 110 million potential NLRP3 inhibitor molecular structures, achieving unprecedented coverage of chemical space [122]. AI-generated molecules still require preclinical and clinical validation and are not assumed to be superior to traditional medicinal chemistry. To date, no AI-designed NLRP3 inhibitor has entered clinical trials for OA, and none has established disease-modifying efficacy in knee OA.

### 3.2. Key Breakthroughs in Druggability Optimization

Translating NLRP3 inhibitors from active molecules to clinical candidates requires systematic breakthroughs in target selectivity and safety. As a localized joint disease, OA necessitates a focus on intra-articular retention and cartilage penetration. It should be emphasized that the clinical candidates discussed in the following sections were primarily developed for non-OA indications; their development progress in other diseases, while validating the druggability of the NLRP3 target, cannot be directly extrapolated to predict their efficacy in knee OA. Each candidate must be independently evaluated in the context of knee OA pathophysiology. No candidate should be assumed to have disease-modifying efficacy in knee OA without dedicated knee OA trials.

### 3.3. Analysis of Clinical-Stage Inhibitors

After years of optimization, NLRP3 inhibitors have entered clinical trials (Table 2). Currently, no NLRP3 inhibitor has entered clinical trials for knee OA; the following section briefly analyzes their development progress in non-OA indications. It should be particularly emphasized that data on clinical-stage NLRP3 inhibitors must be interpreted cautiously according to the hierarchy of evidence, and positive results at any level cannot be directly equated with clinical efficacy. Clinical efficacy must be verified in randomized controlled trials using clinical endpoints such as pain, function, and patient-reported outcomes; structural potential disease modification requires further demonstration of delayed joint structural progression on imaging or histology. To date, no NLRP3 inhibitor has completed validation of the above clinical efficacy or structural modification in knee OA. Therefore, when referring to clinical-stage NLRP3 inhibitors, the terms “efficacy”, “exploratory therapeutic potential”, or “potential disease modification” in this review should be understood as target modulation signals at the preclinical or early clinical stage in non-OA indications, rather than established clinical benefit in knee OA (Table 3).

### 3.4. Preclinical Evidence of NLRP3 Inhibitors in the Context of OA

Although multiple small-molecule NLRP3 inhibitors have entered clinical research for neurological, metabolic, and cardiovascular diseases, no NLRP3 inhibitor has yet entered dedicated clinical trials for knee OA, and current efficacy evidence is almost entirely derived from in vitro cell experiments and rodent OA models. Intra-articular local administration is the most widely investigated intervention modality in the context of knee OA: in DMM surgery-induced mouse OA models, intra-articular injection of MCC950 can inhibit NLRP3 inflammasome activation, suppress chondrocyte pyroptosis, alleviate synovial inflammation, and exert chondroprotective effects; in ACLT-induced OA models, NLRP3 pathway activation contributes to cartilage degeneration, matrix degradation, subchondral bone remodeling, and osteophyte formation, and MCC950 can inhibit these OA pathological changes by targeting NLRP3. Meanwhile, numerous naturally derived NLRP3 modulators, including baicalein and celastrol, have been confirmed in cell and animal models to alleviate joint tissue damage by blocking the NLRP3–caspase-1–GSDMD axis. Intra-articular administration of baicalein dose-dependently reduces OARSI scores in DMM rats, delays OA progression, downregulates inflammatory cytokines IL-1β and TNF-α and degrading enzymes MMP-13 and ADAMTS-5, and reduces type II collagen degradation and the release of CTX-II and COMP; its chondroprotective effect depends on inhibition of the NLRP3 inflammasome and does not affect oxidative stress molecules such as SOD, GSH, and MDA, whereas the positive control dexamethasone improves OA symptoms while also regulating oxidative stress molecules to inhibit the NLRP3 inflammasome [135]. Celastrol, through the SIRT2–NLRP3 axis, upregulates SIRT2 and reduces NLRP3 acetylation, inhibits chondrocyte inflammation and GSDMD-N-mediated pyroptosis, and increases anti-inflammatory factors IL-4 and IL-10; SIRT2 knockdown can counteract its protective effects, and in vivo experiments have also verified that it alleviates OA lesions in rats [136]. The above preclinical results support the theoretical basis for investigating NLRP3 as a candidate target for disease-modification research in OA; however, they do not establish disease-modifying efficacy in human knee OA; however, it must be recognized that a significant gap exists between animal models and human OA pathology.

## 4. Mechanisms and Potential of NLRP3-Targeted Therapy in Knee OA

OA is a highly heterogeneous disease, and the biological effects of NLRP3 differ markedly across pathological subtypes. This chapter focuses on the knee OA-specific pathological mechanisms and intervention strategies. Notably, NLRP3 activation does not play a single role in knee OA: it can act as a pathogenic driver under inflammation- and metabolism-dominated pathological conditions, as a downstream consequence of cartilage and tissue injury responses. Depending on the disease phenotype and stage, it may also represent a combination of these mechanisms. This mechanistic distinction has profoundly different therapeutic implications: if NLRP3 acts only as a secondary inflammatory amplifier after injury, targeted inhibition can only alleviate secondary inflammation and cannot reverse the original pathogenic triggers such as mechanical loading or trauma; only when it acts as a primary driver may it be more likely to have disease-modifying potential; this remains to be tested in dedicated clinical studies.

### 4.1. Pathological Roles of NLRP3 in Knee OA

#### 4.1.1. Chondrocyte Injury and Matrix Degradation

Chondrocytes are the only resident cell type in articular cartilage, and maintenance of their functional homeostasis is critical for the balance between synthesis and degradation of the extracellular matrix (ECM) [139]. During the pathological progression of knee OA, aberrant activation of the NLRP3 inflammasome in chondrocytes mediates pyroptosis and may promote progressive cartilage loss. Studies have shown that inhibiting NLRP3 inflammasome-mediated chondrocyte pyroptosis helps to maintain ECM homeostasis and reduce chondrocyte inflammation; this conclusion is mainly derived from animal models and in vitro experiments [140,141,142].

From the perspective of pathological outcomes, chondrocyte pyroptosis induced by NLRP3 pathway activation further leads to an imbalance between ECM synthesis and degradation. In knee OA cartilage tissue, this process is associated with upregulated expression of matrix-degrading enzymes, accelerating the degradation of type II collagen and aggrecan and ultimately resulting in structural destruction of cartilage [143,144,145]. This mechanism is further supported by intervention studies: Fpr3 gene knockout can alleviate anterior cruciate ligament transection-induced knee OA by inhibiting NLRP3 inflammasome activation, reducing chondrocyte pyroptosis, and attenuating ECM degradation [146]. These findings suggest that NLRP3 inflammasome activation in chondrocytes may be a potential intervention node for cartilage destruction in knee OA, but its causal role in humans still requires further validation.

#### 4.1.2. Synovial Inflammation and Macrophage Polarization Imbalance

Macrophages in synovial tissue are important participants in the early inflammatory responses of knee OA [147]. The expression of NLRP3, caspase-1, and IL-1β is elevated in synovial tissues from patients with OA and experimental OA models. Persistent activation of NLRP3 in synovial macrophages is associated with the release of IL-1β and IL-18, which may induce synovial hyperplasia and chronic inflammation and further damage neighboring chondrocytes in a paracrine manner [148,149,150,151]. This conclusion is mainly derived from human observational studies and animal models.

The polarization state of synovial macrophages plays an important role in the inflammatory regulation of knee OA. Under stimulation by microenvironmental signals, macrophages can polarize into two major phenotypes: a pro-inflammatory (M1) phenotype and an anti-inflammatory (M2) phenotype. Under normal conditions, synovial macrophages maintain a dynamic balance between M1 and M2; however, under the pathological state of knee OA, the M1/M2 ratio becomes imbalanced, with M1 polarization predominating and releasing large amounts of pro-inflammatory cytokines. It should be noted that the M1/M2 classification is a simplified in vitro model, whereas in vivo OA synovial macrophages exhibit multiple transcriptional and functional states with high heterogeneity and dynamic plasticity; therefore, M1/M2 imbalance should be understood as a generalized description rather than a strict dichotomy [152,153]. The key role of synovial macrophage polarization imbalance in OA inflammatory regulation was further validated in pharmacological studies with glibenclamide. Studies with glibenclamide suggest that inhibiting the MAPK pathway and NEK7-NLRP3 interaction can reduce synovial macrophage infiltration, promote M1-to-M2 switching, decrease IL-1β and TNF-α, and restore IL-10; in a co-culture system, it enhanced chondrocyte ECM synthesis, proliferation, and migration. Conditional knockout of NLRP3 in synovial macrophages attenuated the protective effects of glibenclamide, suggesting that its function depends on macrophage NLRP3 [137,138]. The above evidence suggests that targeting the NLRP3 pathway in synovial macrophages may help rebalance M1/M2, alleviate synovial inflammation and secondary cartilage damage in knee OA, but its specific mechanisms and early translational research value still require further investigation.

#### 4.1.3. Subchondral Bone and Mechanotransduction

Subchondral bone remodeling runs throughout the pathological process of knee OA, with typical changes including subchondral bone sclerosis, osteophyte formation, and bone marrow edema-like lesions. The subchondral bone microenvironment is not an effector field of a single cell type; osteoblasts, osteoclasts, osteocytes, and bone marrow MSCs can all express NLRP3 inflammasome components and jointly participate in osteoblast–osteoclast coupling imbalance [154]. Persistent activation of NLRP3 in osteoclasts can promote RANKL secretion through the caspase-1/IL-1β axis, driving osteoclast overactivation and enhanced bone resorption [155]; aberrant activation of NLRP3 in osteoblasts may inhibit osteogenic signaling pathways such as Wnt/β-catenin, weakening bone formation capacity [156]; NLRP3 activation in osteocytes and bone marrow MSCs may also affect the conversion of mechanical loading into bone remodeling signals and their osteogenic differentiation potential, further exacerbating osteoblast–osteoclast imbalance [155].

Analysis of clinical specimens suggests that the expression levels of NLRP3, ASC, caspase-1, and IL-1β in the subchondral bone of patients with knee OA are elevated compared with normal controls, and their intensity is positively correlated with the degree of subchondral bone sclerosis and osteophyte size [157]. However, limited by the difficulty in obtaining human bone tissue samples and ethical constraints, current conclusions are mostly based on tissue homogenate detection, making it difficult to precisely distinguish specific cellular sources and intercellular interactions; therefore, human tissue evidence currently remains at the level of associative observation.

Multiple OA animal models provide more direct evidence for the above mechanisms. In DMM- and ACLT-induced OA models, inhibition of NLRP3 (via gene knockout or MCC950) attenuates subchondral bone sclerosis and suppresses osteophyte formation; its effects on osteoclasts may vary by model, but the overall trend is modulation of abnormal bone remodeling [137]. In MIA-induced OA models, NLRP3 activation is closely associated with subchondral bone marrow lesions [158]. However, it should be noted that the pathological drivers differ significantly among animal models, and extrapolating conclusions from animal models to human OA requires full consideration of their mechanistic heterogeneity.

**Figure 3:**
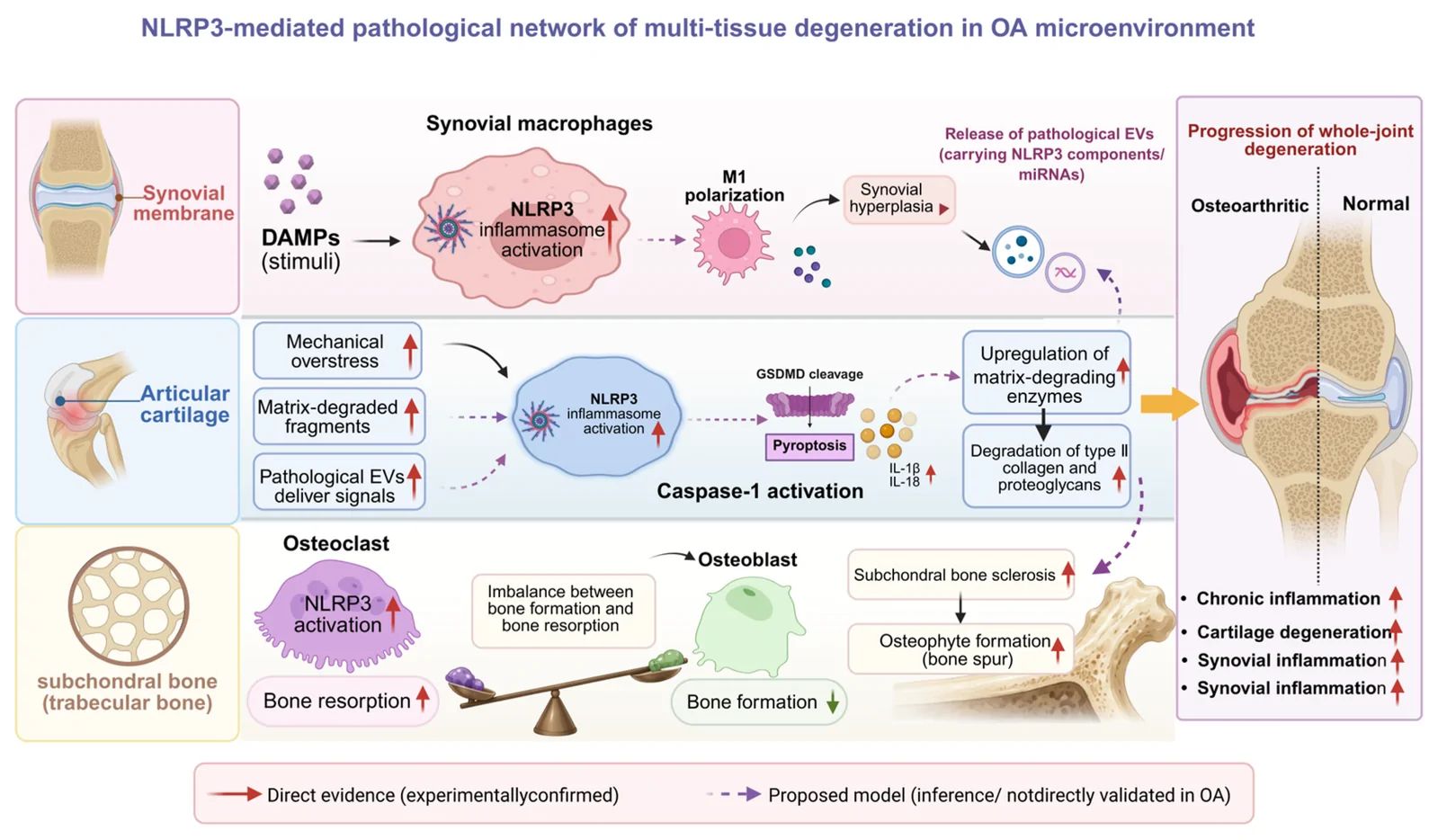
NLRP3-mediated pathological network of multi-tissue degeneration in the OA microenvironment. This schematic illustrates a proposed interactive degenerative network mediated by the NLRP3 inflammasome within the knee OA microenvironment. In the cartilage layer, NLRP3 activation may mediate chondrocyte pyroptosis and upregulate matrix-degrading enzymes, thereby accelerating the degradation of type II collagen and aggrecan. In synovial tissue, NLRP3 may drive macrophage polarization toward the pro-inflammatory M1 phenotype, promoting the release of IL-1β, TNF-α, and other inflammatory cytokines, which may induce synovial hyperplasia and chronic inflammation and further damage adjacent chondrocytes via paracrine effects. In the subchondral bone region, aberrant NLRP3 activation may disrupt the osteoblast–osteoclast balance, leading to subchondral bone sclerosis and osteophyte formation. Red solid arrows indicate the mechanisms supported by direct experimental evidence, mainly from in vitro and animal studies; purple dashed arrows indicate proposed, inferred, or not-yet-directly-validated mechanisms in knee OA, including EV-mediated trans-tissue propagation of inflammatory signals and the proposed progression from focal injury to pan-articular degeneration. Importantly, the multi-tissue pathological network of NLRP3 in knee OA is currently based mainly on preclinical evidence, and direct causal validation in humans remains limited; the functional relevance of EV-mediated NLRP3 signal propagation in humans still requires confirmation.

Regarding whether NLRP3 inhibition alters bone remodeling independently of cartilage effects, current evidence is insufficient and controversial. On the one hand, matrix degradation fragments and DAMPs released from cartilage degeneration can diffuse to the subchondral bone via synovial fluid, activating NLRP3 in osteocytes and osteoclasts; this represents a “cartilage-dependent” alteration in bone remodeling [159]. On the other hand, in cartilage-specific NLRP3 knockout models, subchondral bone sclerosis is not completely alleviated, suggesting that local NLRP3 activation in bone tissue may have a pathological contribution independent of cartilage [160]. Furthermore, osteocyte-specific NLRP3 knockout can partially improve DMM-induced subchondral bone remodeling but cannot fully reverse cartilage degeneration, suggesting complex bidirectional crosstalk of NLRP3 between cartilage and subchondral bone. Future studies with more tissue-specific gene knockout or conditional intervention are needed to clarify the independent pathogenic role of NLRP3 in bone remodeling (Figure 3).

Mechanotransduction is an important link connecting mechanical loading to NLRP3 activation. Mechanical loading can indirectly activate NLRP3, rather than directly activating it, through Piezo1 activation, calcium signaling disturbance, mitochondrial dysfunction/ROS generation, and NF-κB priming [161]. This pathway converts mechanical stress into inflammatory signals and may have important pathological significance in knee OA. This model is mainly based on animal and in vitro experiments, and direct human evidence remains limited (Table 4).

### 4.2. Challenges and Unresolved Issues

Despite encouraging preclinical evidence, early translational research of NLRP3-targeted therapy in OA still faces numerous challenges. The effects of long-term local administration on chondrocyte physiological function and joint immune defense remain unclear, whereas intra-articular injection is limited by rapid drug clearance and short retention within the joint cavity [173,174]. The heterogeneity of OA suggests the need to establish biomarker-based patient stratification strategies. Currently, evidence supporting NLRP3 as a potential candidate target for disease-modification research in knee OA is mainly derived from preclinical studies, and human clinical evidence remains lacking; long-term safety, optimization of local delivery, and patient stratification are key unresolved issues on the path to early translational research.

#### Conflicting Evidence and Boundary Conditions for NLRP3 in Knee OA

Although most preclinical and ex vivo studies support a pathogenic role for the NLRP3 inflammasome in knee OA, the literature is not entirely consistent. Some studies have reported that stress-induced cartilage degradation or joint inflammation can occur independently of NLRP3, suggesting that its role is “context-dependent” rather than absolute. For example, Bougault et al., using human osteoarthritic cartilage samples and mouse models, found that mechanical compression could independently induce glycosaminoglycan loss and matrix-degrading enzyme activation in cartilage explants under NLRP3-deficient conditions, indicating that stress-induced cartilage degradation does not necessarily depend on the NLRP3 inflammasome [175]. Consistent with this, multiple studies have reported that NLRP3 gene knockout or pharmacological inhibition fails to consistently delay disease progression, with some experiments even observing no phenotypic difference or only marginal improvement, suggesting that NLRP3 may not constitute an essential node in all joint degeneration pathways.

These discrepancies are more likely to arise from heterogeneity in experimental conditions and disease biological context rather than from purely methodological error. The following six levels of variables are particularly critical.

(1) Model-dependent differences in upstream signals. Different animal models of OA apply distinct types of pathological stress to the joint, and the upstream signaling cascades activated also differ: Destabilization of the medial meniscus (DMM) primarily models meniscal destabilization and subsequent chronic mechanical remodeling; ACLT is dominated by mechanical instability following acute ligament rupture; MIA induces chondrocyte metabolic crisis and rapid cell death by inhibiting glycolysis, with synovial inflammation being a secondary phenomenon; and direct mechanical loading models apply controlled compressive or shear forces [176,177]. The extent of NLRP3 pathway involvement therefore varies significantly among these models, and positive results from a single model cannot be directly extrapolated to other models or to human knee OA. This partly explains why conclusions regarding whether NLRP3 is an essential node vary with model selection.

(2) Temporal effects of disease stage. The functional role of NLRP3 may shift as the disease progresses, gradually transitioning from a primary pathogenic driver to a secondary inflammatory amplifier. Early lesions are characterized by predominant inflammatory signaling and limited structural damage, where NLRP3 is more likely to be positioned upstream of pathology; advanced lesions are accompanied by extensive cartilage loss, subchondral bone remodeling, and mechanical mismatch, where matrix degradation can be completed through NLRP3-independent pathways driven purely by biomechanical factors. Gang Xu et al., using a DMM mouse model, found significant differences in treatment effects across different treatment durations [178], and Yu Li et al., in a rat MIA model study, also demonstrated that NLRP3/GSDMD/IL-1β was significantly inhibited only in early synovium [179]. This stage-dependent dichotomy suggests that NLRP3-targeted intervention has more consistent efficacy in prophylactic or early-stage models, but limited benefit in late-stage models with established structural damage. Accordingly, a testable prediction can be proposed: the efficacy of the same intervention should decline as the administration time point is delayed. The trend in the existing literature that early intervention is superior to delayed intervention is consistent with this, but systematic head-to-head time-course studies are still lacking for confirmation.

(3) Heterogeneity of cell subpopulations. NLRP3 function differs significantly among different joint cells. A review by Yang et al. [141] pointed out that chondrocytes, synovial fibroblasts (FLS), and subchondral bone cells exhibit varying degrees of dependence on NLRP3. Du et al. [180] found that cartilage lysates (CLs) are internalized by FLS, and their protein LPP activates the NLRP3 inflammasome in FLS by inhibiting CAV1, driving pyroptosis, rather than being predominantly driven by chondrocytes themselves. This highlights the heterogeneity of intercellular signal transduction. Measuring total NLRP3 activity in mixed tissue preparations can easily be diluted by low-responding subpopulations, leading to contradictory efficacy results; therefore, targeted intervention requires clear identification of the dominant cell subpopulation and stratified evaluation.

(4) Differences in patient inflammatory and metabolic phenotypes. Knee OA phenotypes characterized by obesity-related metabolic abnormalities or hyperuricemia more strongly support persistent NLRP3 activation: in high-fat diet-fed mice, increased xanthine oxidoreductase (XOR) activity in infrapatellar fat pad (IPFP) is accompanied by upregulation of NLRP3, iNOS, and IL-1β and promotes cartilage lesions, and febuxostat inhibits this axis. Xanthine oxidoreductase activation is implicated in the onset of metabolic arthritis [181]; in human knee OA synovial fluid, uric acid is positively correlated with IL-1β and IL-18, and is associated with radiographic/bone scan severity and 3-year changes; in non-gout medial knee OA, baseline serum uric acid predicts 24-month medial joint space narrowing [182]. Elevated NLRP3/ASC expression can be observed in synovial tissue from human total knee replacement [157]. The above inflammatory/metabolic subpopulations may serve as stratified populations for NLRP3 targeting; mechanical or aging-dominant phenotypes are relatively less dependent on NLRP3, and lack of stratification will confound efficacy conclusions.

(5) Mechanical loading can act independently of the NLRP3 pathway. Varus deformity, increased medial compartment contact force, and elevated dynamic knee adduction moment can initiate cartilage catabolism through mechanosensitive ion channels and cytoskeleton-nuclear signal transduction [183]. A recent annual review of OA biology classified TRPV4/PIEZO1, cytoskeletal regulators, and YAP/TAZ-related mechanical responses as a relatively independent “mechanome”, whose abnormalities can regulate matrix metalloproteinases, aggrecanases, and chondrocyte dedifferentiation without necessarily using NLRP3 as the sole hub [184,185]. In OA, Piezo1 can directly promote matrix degradation via Ca^2+^ influx, NF-κB/MAPK, and ferroptosis-like responses, and can also activate NLRP3 secondarily through mitochondrial damage and cGAS-STING under sustained abnormal loading, suggesting that mechanical driving and inflammasome activation may be in an “upstream-downstream” or “parallel dual-track” relationship [186,187]. Therefore, in mechanically dominant subtypes, inhibiting NLRP3 alone may be insufficient to block progression; load reduction (orthopedic alignment correction, weight loss, gait/adduction moment retraining) simultaneously improves cartilage damage and secondary inflammation, and is more consistent with the pathological sequence of this subtype.

(6) Methodological variation. Caspase-1 cleavage reflects assembly and zymogen activation, GSDMD-N reflects pyroptotic execution, mature IL-1β/IL-18 reflects downstream cytokine output, and ASC specks reflect adaptor polymerization; these four are not equivalent in time course or cell type, and reading only one of them can create apparent discrepancies between “activated” and “not activated” [188,189]. At the sample level, human synovial fluid directly reflects the local joint environment but is affected by aspiration volume, viscosity, dilution, and blood contamination; peripheral blood is easy to sample repeatedly but is influenced by systemic metabolic/inflammatory status; and there is a lack of unified comparable standards among cartilage, synovium, primary cells, and mouse DMM/ACLT models, with strain, age, sex, and modeling intensity further amplifying heterogeneity [185]. In EV studies, preparations obtained by differential ultracentrifugation, size-exclusion chromatography, polymer precipitation, ultrafiltration, and immunoaffinity differ systematically in purity, yield, and cargo composition—ultracentrifugation can yield relatively pure small particles but tends to aggregate, precipitation co-pellets lipoproteins/aggregates, SEC better preserves structure but may lose very small components, and affinity capture yields highly pure subpopulations but depends on antibodies and pre-processing; therefore, differences in functional interpretation across studies may arise from isolation rather than biological opposition [190,191]. It is recommended to report source, isolation workflow, positive/negative markers, orthogonal particle sizing, and lipoprotein/protein aggregate contamination according to MISEV2023.

In summary, these contradictory findings do not negate the role of NLRP3 in specific OA subtypes; rather, they reinforce the core argument of this review: NLRP3 targeting is most likely to be applicable to the biologically defined subset of OA patients with inflammation- and metabolism-dominant phenotypes, rather than representing a universal disease-modifying strategy for all patients with knee OA. Recognizing this heterogeneity is not a limitation but a prerequisite for designing clinically meaningful trials: future NLRP3 inhibitor trials should incorporate biomarker-based patient stratification strategies to enrich the most likely responders, rather than enrolling unselected OA cohorts in which treatment effects may be diluted by NLRP3-independent disease subtypes.

## 5. EVs: A New Dimension in Diagnosis and Therapy of NLRP3-Related Diseases

EVs are membrane-based vesicular structures secreted by cells that mediate intercellular information transfer by encapsulating active molecules such as proteins, lipids, and nucleic acids. According to the current International Society for EVs framework, when endosomal origin has not been clearly demonstrated, “EVs” should be used uniformly; small vesicles can be described as “small extracellular vesicles (sEVs)”; only when the original study clearly identifies exosomal markers such as CD63, CD81, and TSG101 and has electron microscopy morphological evidence may “exosomes” be used [192]. In the pathological process of OA, EVs serve both as paracrine mediators that transmit inflammatory signals among synovium, cartilage, and subchondral bone, amplifying NLRP3-mediated pyroptosis and matrix degradation, and as engineered tools for precise modulation of the NLRP3 pathway. Meanwhile, the pathway-specific molecules carried by EVs hold potential as biomarkers for disease stratification and efficacy assessment. This chapter systematically elaborates on the interactions between EVs and the NLRP3 pathway from four perspectives: molecular regulatory mechanisms, exploratory biomarker applications, investigational therapeutic strategies, and translational bottlenecks, and focuses on defining the current boundaries of evidence.

### 5.1. Bidirectional Molecular Mechanisms of EV-Mediated Regulation of NLRP3 Activation

#### 5.1.1. Pathway Mechanisms by Which Pathological EVs Promote NLRP3 Hyperactivation

EV preparations released by damaged cells and activated immune cells under pathological conditions have been reported to contain molecules capable of triggering NLRP3 inflammasome activation at multiple levels, suggesting a potential inflammatory cascade amplification effect. However, functional EV-mediated transfer requires rigorous demonstration and cannot be inferred from cargo detection alone. At the priming stage, DAMPs such as HMGB1 and HSP70 displayed on EV surfaces engage TLR4 on recipient cells. This triggers downstream NF-κB signaling and upregulates transcription of core inflammasome components (e.g., NLRP3, pro-IL-1β), establishing molecular prerequisites for subsequent inflammasome assembly [193,194,195,196]. At the activation signal level, EVs may trigger NLRP3 activation through the following pathways: first, ATP carried by EVs activates the P2X7 receptor on target cell membranes in a paracrine manner, inducing intracellular potassium efflux [197,198]; second, studies have shown that chondroitin sulfate and collagen degradation fragments carried by EVs, upon phagocytosis by macrophages, induce lysosomal membrane rupture and the release of cathepsin B, which directly activates NLRP3 [199,200,201,202,203].

In the OA microenvironment, EV-mediated signal transmission may constitute a multi-tissue interactive pathological network. Shao et al. reported that NLRP3-positive EV preparations released by synovial macrophages could penetrate the superficial cartilage matrix and, upon uptake by deep-zone chondrocytes, induce pyroptosis and secretion of matrix-degrading enzymes in preclinical models. However, this mechanism has not been directly validated in human OA, and the functional contribution of EV-mediated transfer requires further confirmation. In addition, studies have reported that miR-16-5p carried by chondrocyte-derived apoptotic vesicles may regulate the TGF-β pathway and potentially modulate the NLRP3 pathway to improve the joint microenvironment and bone remodeling. However, these findings remain preclinical and require functional validation. This cross-tissue signal transmission mode is considered an important candidate mechanism for the progression of OA from local inflammation to pan-articular degeneration. However, it must be emphasized that the above evidence mainly comes from in vitro cell experiments and animal models, and whether EVs in humans directly propagate NLRP3 inflammasome activation in the same manner still lacks direct functional verification.

#### 5.1.2. Multi-Target Regulatory Network of EVs Investigated as Therapeutic Vectors in Suppressing NLRP3 Activation

EVs derived from immunomodulatory cells such as MSCs can suppress NLRP3 activation at multiple levels, including transcriptional regulation, post-translational modifications, and upstream signal blockade. At the post-transcriptional regulatory level, microRNAs carried by EVs can precisely target key nodes of the pathway: miR-146a targets interleukin-1 receptor-associated kinase 1 (IRAK1) and TNF receptor-associated factor 6 (TRAF6), blocking NF-κB-mediated NLRP3 transcriptional upregulation [195,204,205]; miR-223 directly binds to the 3′-untranslated region (3′-UTR) of NLRP3 messenger RNA (mRNA), inhibiting its protein translation [206]; studies have found that BMSC-derived EVs can deliver miR-223-3p to directly target and inhibit NLRP3, attenuating inflammasome activation and promoting mitophagy, thereby alleviating renal ischemia–reperfusion injury [207]; at the post-translational modification level, sirtuin 1 (SIRT1) carried by EVs can catalyze deacetylation of lysine residues on the NLRP3 protein, inhibiting its oligomerization activity [208,209].

In addition, EVs investigated as therapeutic vectors can suppress NLRP3 activation by blocking upstream triggering events. The mitochondrial protective factors they carry can improve mitochondrial respiratory function and reduce mtROS production; lysosomal membrane-stabilizing proteins can maintain lysosomal integrity and prevent cathepsin release. Meanwhile, EVs can regulate macrophage metabolic reprogramming, promoting the switch from glycolysis to oxidative phosphorylation, reducing cellular metabolic levels, and suppressing the energy supply for NLRP3 activation. This multi-node, multi-level regulatory mode gives EVs investigated as therapeutic vectors potential anti-inflammatory advantages in complex inflammatory microenvironments. For example, mesenchymal stem cell-derived EVs in intervertebral disc degeneration and spinal cord injury models can deliver miR-146a to target the IRAK1/TRAF6/NF-κB axis, downregulating NLRP3 transcription while reducing mtROS production to block activation signals [210,211]. However, the above results still mainly constitute preclinical evidence, and their human efficacy and safety have not yet been established.

#### 5.1.3. Methodological Critique and Boundaries of Causal Inference

Current research on the regulation of NLRP3 by EVs faces significant methodological challenges. First, EV preparations may simultaneously contain EV-associated proteins, soluble protein contamination, membrane-bound proteins, luminal cargo, proteins released due to pyroptosis, protein aggregates, or non-EV particles [192,212]. Isolation techniques such as ultracentrifugation, size-exclusion chromatography, and affinity capture do not necessarily yield pure EV preparations [213,214]. Therefore, in the absence of topological characterization, removal of free components, and calibration of particle quantification, detected NLRP3, caspase-1, or IL-1β signals cannot be directly equated with EV luminal cargo or EV-mediated functional transfer [215,216,217,218].

Second, demonstrating that “inflammatory molecules are functionally transferred to recipient cells via EVs” requires more rigorous experimental design, including characterization of EV markers and negative markers, density-gradient or immunoaffinity purification, protease protection assays, detergent treatment controls, single-particle characterization, particle concentration and size distribution, cargo quantification, recipient cell functional responses, and blocking experiments [196,219,220]. Co-localization or co-isolation results alone are insufficient to rule out the influence of soluble factor contamination or products released during pyroptosis.

Therefore, statements that “EVs carrying NLRP3 components propagate inflammation” should be strictly qualified: current in vitro and animal-model evidence supports the biological plausibility of this mechanism and its possible involvement in inflammatory amplification in knee OA; however, direct functional validation in humans is still lacking, and the causal contribution, cellular source, target cell type, and disease-stage dependence of this mechanism require further investigation. Detection of a protein or RNA within an EV preparation does not prove functional EV-mediated transfer; demonstration of cargo association, cellular uptake, and biologically relevant functional transfer represents a substantially higher level of evidence.

### 5.2. EVs as Biomarkers for NLRP3-Related Diseases

#### 5.2.1. Molecular Basis and Detection Advantages of EV-Based Biomarkers

Free circulating NLRP3 pathway molecules in body fluids are susceptible to protease-mediated degradation, with short half-lives and poor detection stability, making it difficult to accurately reflect local tissue-specific pathway activation. The phospholipid bilayer of EVs effectively protects encapsulated molecules, maintaining their structural and functional stability in body fluids over extended periods, and enables enrichment and high-sensitivity detection through density gradient centrifugation or immunocapture. The pathway components carried by EVs are highly correlated with the activation state of their parent cells, and can truly reflect the inflammatory status of the source tissues; combined with immunocapture techniques targeting tissue-specific membrane proteins, EVs from different cellular sources can be separately detected, enabling precise assessment of tissue-specific NLRP3 activation levels. However, EVs as biomarkers still face issues such as the lack of unified standards for pre-analytical processing, isolation and enrichment, particle quantification, and protein and nucleic acid detection platforms. Detection thresholds, normalization methods, and result comparability among different studies are limited. Therefore, the detection advantages of EVs should currently be described as “potential advantages” rather than established clinical diagnostic capabilities.

#### 5.2.2. Diagnostic and Prognostic Stratification Value in OA

Zhang et al. [221] performed a comprehensive proteomic characterization of matched plasma and synovial fluid EVs from a knee OA proteomic cohort (n = 16). EVs were isolated by polymer-based precipitation (ExoQuick), and proteomic profiling was performed using mass spectrometry. The study identified 692 synovial fluid EV peptides positively associated with knee radiographic OA severity (K/L, JSN, OST), with 57.4% derived from immune system proteins. Surface markers including CSPG4, BGN, NRP1, and CD109 were identified and further validated in OA joint tissues by scRNA-seq (n = 3) and in synovial fluid by high-resolution flow cytometry (n = 23). However, these findings remain exploratory and lack independent external validation. Another study by Zhang et al. [222] evaluated CSPG4+ plasma EVs in a discovery cohort (n = 51) and an independent validation cohort (n = 92) of knee OA patients, with additional synovial fluid analysis (n = 61). EVs were isolated by 100 kDa ultrafiltration and characterized by high-resolution flow cytometry and ELISA. Plasma CSPG4+ EV frequencies were positively associated with radiographic OA severity (K/L, JSN) but not with symptomatic severity (WOMAC, KOOS pain). In synovial fluid, CSPG4+ EVs were enriched in fibronectin (FN1) and interleukin-11 (IL-11). Although the association between plasma CSPG4+ EV frequency and radiographic severity was replicated in an independent cohort, this evidence remains associative and lacks prospective clinical validation or demonstration of diagnostic utility. The review by Li et al. [223] is not a primary validation study but emphasizes that multicenter prospective cohorts, standardized EV isolation and detection platforms, and independent external validation are needed before EV-based biomarkers can be considered clinically useful. Importantly, association with disease severity is not equivalent to diagnostic validity, and an elevated area under the receiver operating characteristic curve (AUC) or correlation in an exploratory cohort does not establish clinical utility, external validity, or superiority over established biomarkers. At present, no EV-based biomarker has been prospectively validated for diagnosis, stratification, or treatment monitoring in knee OA.

### 5.3. Engineered EVs for NLRP3-Targeted Investigational Therapeutic Strategies

The anti-inflammatory activity of naïve EVs is limited, and engineering modifications can enhance their NLRP3-targeting regulatory capacity, improving the precision and efficacy of local therapy in OA. Via techniques such as electroporation and sonotransfection, cargo engineering enables the loading of NLRP3 siRNA, PYD-domain peptide inhibitors or small-molecule inhibitors into EVs to block NLRP3 activation at multiple layers, including gene expression, protein interactions, and pathway activation [206,224]; in addition, construction of co-delivery systems simultaneously carrying NLRP3-regulatory molecules and MMP inhibitors can achieve dual effects of inflammation suppression and matrix protection. Studies have shown that MSC-derived EVs electroporated with NLRP3 siRNA achieved higher gene silencing efficiency compared to free siRNA, with an increased inhibition rate of IL-1β secretion [225].

Surface engineering modifications can enhance lesion enrichment efficiency through targeting ligand decoration: the type II collagen-binding peptide (WYRGRL) can mediate specific anchoring of EVs to cartilage matrix, enhancing deep cartilage penetration and joint retention; fibroblast activation protein (FAP) antibodies enable targeted enrichment to activated synovial fibroblasts; hyaluronic acid modification enhances target cell EV uptake via CD44 receptor engagement. Studies on targeting peptide-modified EVs have demonstrated that type II collagen peptide modification can increase cartilage enrichment efficiency by approximately 3-fold, with joint retention time exceeding 7 days, potentially enhancing the inhibitory effect on the NLRP3 inflammatory pathway at the lesion site [226,227]. Beyond targeting modifications, the introduction of ROS-sensitive chemical bonds such as disulfide bonds and boronate esters into EV membranes enables the construction of inflammatory microenvironment-responsive „smart” EVs that achieve lesion-directed drug release, increasing drug concentration at inflammatory sites while reducing drug accumulation in normal tissues. Based on this concept, ROS-responsive carriers loaded with the NLRP3 small-molecule inhibitor MCC950 have been shown to significantly prolong local drug action duration in the joint, simultaneously alleviating synovial inflammation and correcting abnormal subchondral bone remodeling while reducing systemic toxicity, as summarized from multiple joint-targeted nanodrug delivery studies [117,228,229].

Overall, engineered EVs are investigational delivery vectors with potential application value, rather than an established clinical therapeutic platform. No engineered EV product has demonstrated disease-modifying efficacy in knee OA. Their early translational research still needs to address issues such as loading efficiency, modification uniformity, targeting specificity, in vivo fate, and long-term safety.

### 5.4. Core Bottlenecks and Optimization Directions for Early Translational Research

Standardization of preparation processes and quality control constitutes the primary obstacle to early translational research. Existing isolation techniques, including ultracentrifugation and size-exclusion chromatography, generate preparations with substantial inter-batch variability, and process fluctuations during large-scale manufacturing (e.g., cell culture and cryopreservation) readily lead to inconsistent product quality [230]. It is necessary to establish unified isolation and purification workflows and to define core quality control parameters, including particle characterization, size distribution, concentration, morphology, classical positive markers, negative/non-EV markers, protein contaminants, aggregation status, inter-batch variability, source cell heterogeneity, culture conditions (including serum-containing and serum-free media), isolation methodology, storage conditions, freeze–thaw effects, and methods for cargo quantification and potency testing. For engineered EVs, loading efficiency and modification homogeneity must also be quantified to ensure batch-to-batch consistency [231].

In vivo delivery efficiency and safety evaluation systems remain incomplete. Following systemic administration, EVs are largely cleared by the reticuloendothelial system of the liver and spleen; after intra-articular administration, they readily enter the circulation via lymphatic drainage, resulting in limited local retention time, and their in vivo distribution and metabolic profiles still lack systematic in vivo tracing data [232]. The intra-articular retention, cartilage matrix penetration, target cell uptake, and cargo release kinetics of engineered EVs all require further quantitative investigation.

Regarding safety, the complement activation, thrombotic risk, and long-term immunogenicity of xenogeneic EVs require verification, and the lack of long-term toxicity data in large animals constrains regulatory submissions. In particular, it should be noted that a study reported that intravenous infusion of large EVs derived from human umbilical cord MSCs can induce dose- and tissue factor-dependent lethal pulmonary thromboembolism in mice. This safety signal suggests that systemic administration of EVs, especially high-dose intravenous infusion, carries a non-negligible thrombotic risk. In contrast, intra-articular local administration can significantly reduce systemic exposure and offers a potential safety advantage; however, local administration may still pose risks such as synovial irritation, infection associated with repeated injections, effects of local high-concentration exposure on chondrocyte physiological function, and long-term local immunosuppression. Intra-articular administration is not zero-risk; rather, it shifts the safety boundary issue from systemic immunosuppression to local tolerability and organ-specific toxicity, the latter of which likewise lacks support from large-sample long-term data.

Regulatory pathways and translation strategies still require exploration. EVs fall under advanced therapy medicinal products, and global regulatory frameworks remain unstandardized. For OA, priority can be given to developing locally administered autologous EV products to reduce safety risks; alternatively, leveraging the clinical foundation of already marketed mesenchymal stem cell products can facilitate homologous EV translation. Currently, several mesenchymal stem cell-derived EV products have completed Phase I safety evaluations for OA, demonstrating good tolerability and chondroprotective trends, although Phase II efficacy data are still pending [233,234,235] (Table 5).

Overall, EVs offer a new technological dimension for investigating the diagnosis and therapy of NLRP3-related diseases. Their biomarker value and targeted therapeutic potential remain exploratory and have not been fully validated, especially in knee OA. With continued optimization of preparation processes, quality control systems, and delivery technologies, EVs may become the next-generation core technology for NLRP3-targeted therapy, providing novel solutions for precision diagnosis and treatment of chronic inflammatory diseases such as OA.

## 6. Challenges and Future Directions in Clinical Trials

NLRP3 involvement in the pathogenesis of monogenic autoinflammatory diseases (such as CAPS) is supported by substantial genetic and biological evidence, and blockade of the downstream IL-1β/IL-1R pathway has established clinical efficacy in these diseases; however, direct NLRP3 inhibition and downstream IL-1 signal blockade are not therapeutically equivalent, and long-term efficacy and safety data for direct NLRP3 inhibitors remain limited. The translation of NLRP3 inhibitors to complex chronic diseases such as OA still faces multiple bottlenecks, including safety boundaries, dose management, combination strategies, and patient heterogeneity. This chapter discusses these issues from four dimensions: safety and dose management, multi-mechanism combination therapy, OA heterogeneity and patient stratification, and empowerment by cutting-edge technologies.

### 6.1. Safety Risks and Precision Dose Management

NLRP3 is a core sensor of danger signals in innate immunity, and long-term systemic inhibition disrupts immune homeostasis, compromising host defense against fungi and intracellular pathogens [242]. The elderly population, among whom OA is highly prevalent, generally experiences age-associated immune senescence. Long-term drug-related infection hazards and potential adverse impacts on mucosal and cutaneous immunity remain poorly supported by large-scale clinical evidence [243,244].

The first-generation lead compound MCC950 was discontinued from clinical development owing to off-target-mediated dose-dependent hepatotoxicity; it should be emphasized that MCC950 is an experimental probe, and its toxicity profile cannot be automatically extrapolated to structurally distinct next-generation inhibitors, which still require individual evaluation. Nevertheless, for OA requiring long-term intervention, there is an urgent need for validation in large-sample studies of long-term risks such as cumulative hepatorenal toxicity and cardiovascular safety [245,246]. The increased infection risk associated with systemic IL-1β neutralization in the CANTOS trial further suggests a cross-drug class-effect safety signal for long-term inhibition of the NLRP3/IL-1 axis.

Although intra-articular local administration can significantly reduce systemic exposure, it introduces local safety issues specific to knee OA: retention of high-concentration drugs in the synovium can induce synovial irritation and even chemical synovitis; repeated joint puncture itself increases the risk of iatrogenic joint infection; and whether long-term sustained inhibition of local NLRP3 in the joint interferes with normal chondrocyte physiological function and weakens local injury repair and anti-infection defense remains to be systematically evaluated [117,178,247]. Thus, intra-articular administration is not zero-risk; rather, it shifts the safety boundary issue from systemic immunosuppression to local tolerability and organ-specific toxicity, the latter of which likewise lacks support from large-sample long-term data.

The core dilemma in dose design is insufficient target tissue exposure: after systemic administration, intra-articular drug concentrations are only 1–5% of plasma levels, making it difficult to achieve effective inhibitory thresholds, while blind dose escalation amplifies systemic toxicity, creating a therapeutic dilemma between efficacy and toxicity (the mechanistic bottlenecks of local joint delivery are detailed in Section 3.4 and Section 5.4 and will not be elaborated here) [248]. Inter-patient heterogeneity in joint degeneration severity and hepatorenal function further exacerbates pharmacodynamic variability, and existing standardized dosing regimens lack local pharmacokinetic/pharmacodynamic evidence, which is an important reason for the suboptimal efficacy of some drug candidates. Future efforts could explore stratified dose design based on NLRP3 activation levels and EV pathway biomarkers, combined with sustained-release formulations to prolong intra-articular drug retention; however, these approaches are currently at the conceptual stage, and no clinically validated dosing regimen for OA exists yet.

### 6.2. Efficacy Enhancement Through Multi-Mechanism Combination Therapy

The current therapeutic system for OA itself centers on combination intervention: stepwise combination regimens comprising NSAIDs, intra-articular injectable preparations, and acetaminophen have been widely used in clinical practice, accumulating practical experience for local administration modes and long-term safety management. In other disease indications, combination strategies of NLRP3 inhibitors with other drugs have also entered clinical research. For example, the RESOLVE-2 trial of NT-0796 in combination with a GLP-1 receptor agonist, which aims to evaluate the pharmacokinetic compatibility and safety attributes of this drug combination, is currently ongoing, and its results have not yet been published. The above studies may provide a reference for the design of combination therapeutic regimens for OA, but they are not yet sufficient to confirm the therapeutic efficacy of NLRP3 combination strategies in OA.

It should be clarified that all current NLRP3 inhibitor + X combination strategies for OA remain at the preclinical hypothesis stage and have not been validated in OA clinical trials; their potential synergistic effects are supported only by indirect evidence from in vitro or animal experiments. Related hypotheses mainly include: combination with NSAIDs to achieve upstream inflammation suppression superimposed on downstream symptom relief, with the aim of reducing NSAID dosage and related adverse reactions [118,249]; combination with cartilage repair agents to delay disease progression through bidirectional intervention—inhibiting matrix degradation while promoting tissue repair. For patients with a hyperinflammatory phenotype, combination with TNF-α or IL-6 inhibitors requires strict limitation of the applicable population and vigilance against the infection risk associated with multiple immunosuppression.

EVs and NLRP3 small-molecule inhibitors possess inherent mechanistic complementarity: small-molecule drugs can rapidly suppress already-activated inflammasomes, whereas mesenchymal stem cell-derived EVs regulate macrophage phenotypes and promote cartilage matrix synthesis by delivering functional non-coding RNAs [250,251]. Based on this, engineered EVs loaded with NLRP3 inhibitors have been proposed to simultaneously enhance local drug enrichment efficiency and therapeutic activity [224]. All of the above combination regimens await systematic preclinical validation and subsequent confirmation in clinical trials.

### 6.3. OA Heterogeneity and Inflammatory Subtype Stratification

OA is no longer regarded as a homogeneous degenerative joint disease mediated solely by mechanical wear, but rather as a group of highly heterogeneous clinical syndromes driven by the interaction of multiple factors, including mechanical stress, aging, metabolic disturbance, trauma, and genetic susceptibility. According to the core pathogenic drivers, knee OA can be classified into mechanical load-dominant OA, post-traumatic OA, aging-related OA, metabolic OA, synovitis-driven inflammation-dominant OA, and OA subgroups with chronic pain as the predominant phenotype [252,253]. Existing evidence suggests that different pathogenic drivers may activate local NLRP3 inflammasomes in joints through different danger signals and present different predominant cellular sources; however, systematic comparisons of NLRP3 activation profiles across clinical subtypes of knee OA still lack direct evidence [167,254,255]. This pathological heterogeneity also constitutes a core obstacle to the early translational research of NLRP3-targeted therapy: if the significant anti-inflammatory and chondroprotective effects observed in animal experiments are directly extrapolated to all unselected OA patients, clinical trial efficacy is highly likely to be diluted, or even negative results may occur.

In knee OA subgroups with prominent inflammatory features and metabolic abnormalities, the NLRP3 inflammasome may play an important role in promoting pathology. Elevated synovial NLRP3 expression and oxidative stress levels suggest that local inflammation is closely associated with innate immune activation [256,257]. In the joint microenvironment associated with metabolic abnormalities, danger signals such as uric acid, altered fatty acid profiles, and AGEs may participate in inflammasome priming [182,258]; among these, AGEs can promote the expression of NLRP3, ASC, and caspase-1 in human chondrocytes through mitochondrial ROS and thioredoxin-interacting protein [257]. Synovial macrophages and fibroblast-like synoviocytes are important cellular sources of NLRP3-dependent IL-1β and IL-18 release and pyroptosis [182,259]. Activation of the NLRP3/caspase-1/GSDMD axis can not only promote chondrocyte pyroptosis but also be accompanied by depletion of type II collagen and aggrecan [260]. In addition, inflammatory stimulation can induce chondrocyte senescence and SASP and form a feedback amplification loop with the NLRP3 pathway through the NF-κB/IκB-ζ network [261]. Synovium-derived EVs can also transmit inflammatory and catabolic signals to cartilage through pathways such as NF-κB [262]. In preclinical studies, the NLRP3 inhibitor MCC950 can simultaneously alleviate cartilage inflammation, senescence-like changes, and structural destruction, raising the hypothesis that targeting the NLRP3 pathway may have disease-modifying potential in selected OA contexts; this remains unproven in humans. However, its efficacy in patients with clearly defined inflammatory or metabolic knee OA and the specific mechanisms of vesicle-mediated transmission still require further validation.

In the mechanical overload phenotype, varus malalignment of the knee, increased dynamic knee adduction moment, and elevated medial compartment contact force can lead to local cartilage stress concentration, representing important initiating factors for disease onset and progression [263,264]. Existing evidence indicates that mechanical compression can independently induce glycosaminoglycan loss and matrix-degrading enzyme activation in cartilage explants, and this process can still occur under NLRP3-deficient conditions [175]; conversely, reducing mechanical loading can simultaneously alleviate cartilage damage, secondary inflammation, and NLRP3 expression [167]. Therefore, in mechanical load-dominant OA, NLRP3 is more likely to act as a secondary inflammatory amplifier after tissue injury, rather than as the initial driver of mechanical damage. Although targeting NLRP3 alone may attenuate secondary inflammatory responses, it cannot correct abnormal alignment, compartment loading, or cartilage stress distribution, and thus cannot reverse established mechanical matrix destruction.

In advanced OA, large-area cartilage loss, subchondral bone remodeling, and joint structural deformity have already formed, and current disease-modifying strategies cannot effectively reconstruct the depleted cartilage matrix [265]. In addition, in some patients with a chronic pain phenotype, pain is inconsistent with the degree of imaging damage and may be dominated by mechanisms such as central sensitization; in such patients, inhibiting local synovial NLRP3 alone may be insufficient to adequately control pain [252,266].

Because knee OA exhibits significant pathological and clinical heterogeneity, applying NLRP3-targeted intervention to all patients without selection may dilute therapeutic effects and increase the risk of non-response. KL grading mainly reflects structural changes such as osteophyte formation and joint space narrowing and cannot replace direct assessment of synovial inflammation; therefore, it is difficult to identify early inflammatory-type patients based on X-ray alone [267,268]. Contrast-enhanced MRI and standardized synovitis scoring can relatively reliably reflect synovial histological inflammation. When combined with metabolic indicators, EV-derived signature molecules from synovial fluid or peripheral blood, and multi-omics data, these modalities may provide non-invasive or minimally invasive quantitative information on the degree of NLRP3 activation [269,270,271,272]. MRI-based artificial intelligence phenotype recognition models have been able to distinguish different structural phenotypes such as inflammation, bone, meniscus/cartilage, and hyperplasia [273,274]; however, their ability to identify NLRP3-dependent OA populations remains unvalidated. Future studies still need to validate the consistency between EV features and in situ NLRP3 pathway activity in joints in prospective cohorts, and further confirm their association with response to NLRP3-targeted therapy; establishing an actionable inflammatory endotype stratification system remains a proposed research framework, not an established clinical tool, and no such system has been validated for predicting NLRP3-targeted treatment response in knee OA.

At present, the use of EV liquid biopsy for NLRP3 activation stratification still faces significant translational limitations. Sample pre-processing, EV isolation and enrichment, particle quantification, and protein and nucleic acid detection platforms still lack unified standards, and detection thresholds and result comparability across studies are limited [192,275,276]. Existing knee OA studies are mostly single-center exploratory studies with limited sample sizes, unbalanced distributions of patient age, disease stage, and comorbidities, and insufficient independent external validation [221,277]. In addition, EV isolates simultaneously contain membrane-associated proteins, luminal cargo, free proteins, and non-vesicular particulate components; in the absence of topological localization, removal of free components, and particle quantification calibration, measured signals cannot be directly equated with NLRP3 pathway activation burden [213,278].

Future research should establish unified pre-analytical workflows, EV quality control standards, and multi-platform detection specifications in accordance with MISEV2023, clarify methods for distinguishing luminal cargo, membrane-bound proteins, and contaminating components, and determine detection limits, batch correction schemes, and stratification cutoff values through multicenter cohorts [192,279]. Further construction of a translational pathway encompassing EV feature screening, independent validation, patient enrichment and enrollment, and dynamic monitoring during treatment may help establish a candidate companion diagnostic system for predicting response to investigational NLRP3-targeted therapy; however, this closed loop has not yet been established in knee OA and still requires validation through prospective interventional studies [280,281].

### 6.4. Cutting-Edge Technologies Empowering Early Translational Research

Similarly, the application of artificial intelligence in the field of NLRP3 drug development can be divided into four stages: AI-assisted molecular discovery, which relies on generative platforms (such as Chemistry42) to achieve large-scale exploration of chemical space and output candidate molecules, with the representative molecule ISM8969 having received FDA IND approval and entered Phase I clinical research; computational prediction, which uses protein structures to predict the druggability, off-target effects, and safety risks of candidate molecules for upfront optimization of dosing regimens; preclinical validation, in which pharmacodynamic and safety evaluations are conducted in in vitro systems and animal models; and clinical validation, which confirms human efficacy and long-term safety. At present, AI-designed NLRP3 candidate molecules have reached only Phase I, the highest stage of development so far, and the clinical efficacy and long-term safety of AI-generated molecules still require item-by-item experimental validation; it should not be assumed that they are superior to traditional medicinal chemistry development strategies. The exact value of current AI technology is mainly reflected in shortening the molecular discovery cycle and expanding the explorable chemical space.

Patient stratification based on artificial intelligence integrating multi-omics, radiomics, and clinical data (e.g., constructing molecular subtyping models associated with NLRP3 activation to enrich inflammation-dominant populations [246,282]) is currently still an exploratory research direction and has not yet achieved early translational research; whether this subtyping system can be translated into actionable patient selection criteria still awaits validation in prospective cohort studies.

Among other cutting-edge technology directions, the molecular basis and detection value of EV liquid biopsy as a companion diagnostic tool have been systematically discussed in Section 5.2, and its standardization difficulties and stratification application challenges are discussed in Section 6.3 [169,172,277]; novel delivery systems such as responsive hydrogels and cartilage-targeted nanocarriers are expected to overcome the technical bottlenecks of local drug delivery (technical details are described in Section 5.3), but these carrier systems are currently all at the preclinical research stage [283,284,285].

**Figure 4:**
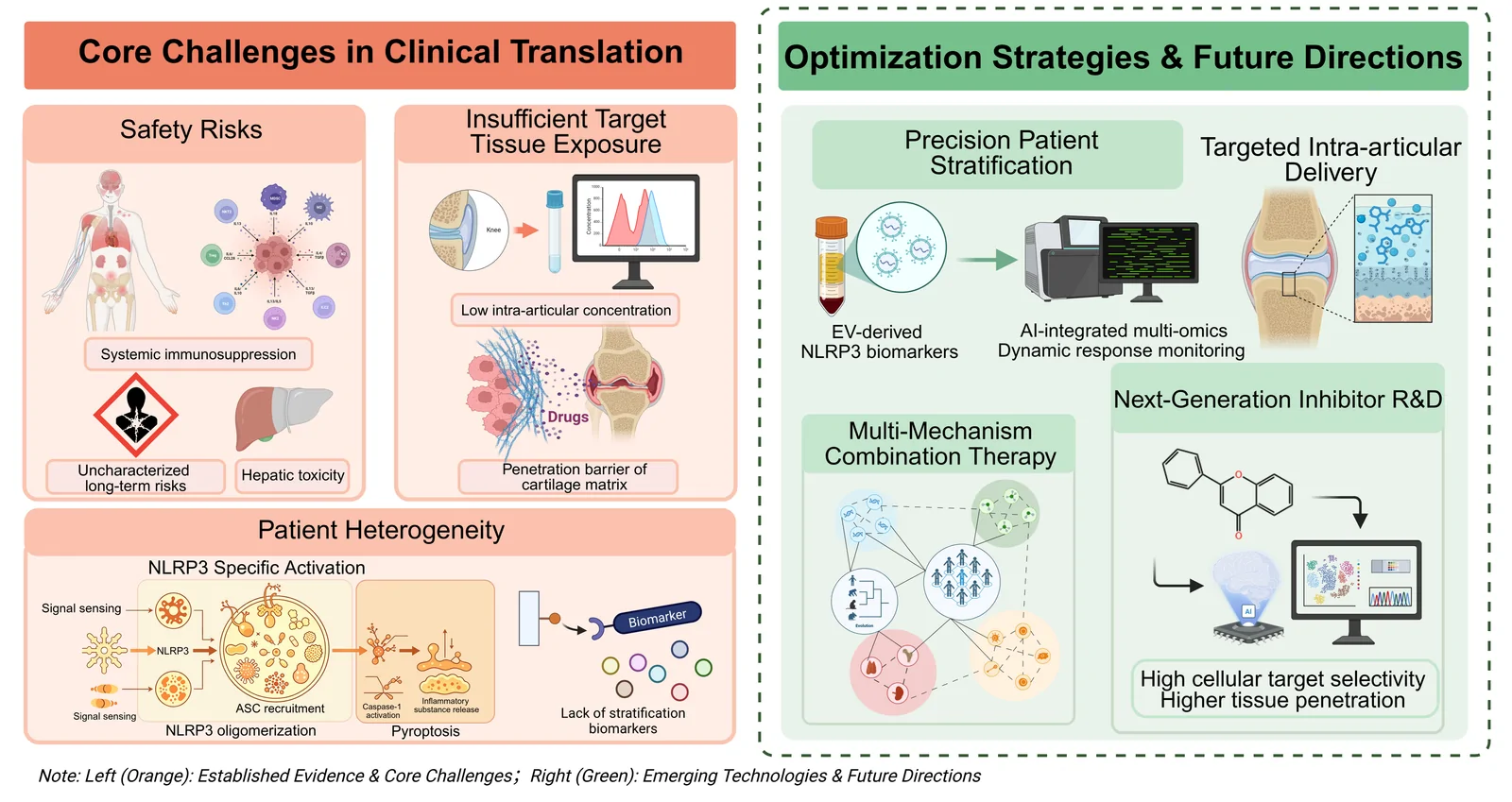
NLRP3 inflammasome inhibitors for OA: early translational research challenges and investigational future directions. This figure summarizes the core challenges in the early translational research of NLRP3 inflammasome inhibitors for OA therapy and the corresponding future optimization strategies. The left orange panels represent currently established evidence and core challenges, including: (1) insufficient target tissue exposure, limited by the cartilage matrix penetration barrier and rapid intra-articular drug clearance; (2) safety concerns, including immunosuppression and hepatotoxicity risks associated with systemic administration, whereas long-term local safety remains to be validated; and (3) marked patient heterogeneity coupled with a lack of precise stratification biomarkers. The right green panels represent emerging technologies and future directions, most of which remain at the preclinical or early exploratory stage and have not yet been clinically validated in OA. These include joint-targeted delivery systems to enhance local drug concentrations; companion diagnostics based on EV-derived NLRP3 pathway molecules to enable patient stratification; AI-driven multi-omics integration to construct inflammatory subtype models; next-generation inhibitors with high selectivity and enhanced tissue penetration; and multi-mechanism combination therapeutic regimens aimed at improving efficacy while mitigating safety risks. This figure should not be interpreted as evidence that these strategies have established clinical efficacy in OA; to date, no NLRP3 inhibitor has entered an OA-specific clinical trial, and EV-based diagnostics and engineered EV therapies remain primarily translational research. Note: Left (orange), established evidence and core challenges; right (green), emerging technologies and future directions, which are not yet clinically validated in OA.

In summary, the early translational research of NLRP3 inhibitors is at a critical stage of expanding toward common complex diseases. In terms of safety management, relatively mature clinical experience with combination therapy and local administration is available for reference; however, trial designs based on patient stratification, multi-mechanism combination intervention strategies, and cutting-edge technologies such as artificial intelligence and EVs are mostly still at the preclinical or early exploratory stage, and their clinical value in improving translation efficiency still needs to be confirmed by prospective clinical trials. Cautiously distinguishing the current status from the prospects is an important prerequisite for improving the success rate of early translational research of NLRP3-targeted therapy (Figure 4).

## 7. Conclusions

This review systematically synthesizes the molecular regulatory network of the NLRP3 inflammasome, the progress in targeted inhibitor development, and the specific pathological mechanisms of NLRP3 in the knee OA microenvironment, as well as EV-mediated diagnostic and therapeutic translational research. The available evidence collectively indicates that the NLRP3 inflammasome is not merely an inflammatory effector element, but one of the important nodes that integrates multiple pathological signals including mechanical overload, metabolic disturbances, senescence-related damage, and organellar stress. NLRP3-mediated chondrocyte pyroptosis, synovial macrophage M1 polarization imbalance, and EV-dependent cascade dissemination of inflammatory signals across cells and tissues may be involved in multiple steps of knee OA progression. This evidence provides a framework for understanding the heterogeneity of knee OA and exploring potential disease-modifying targets, but causal evidence in humans remains limited. Furthermore, it must be clearly recognized that the clinical development progress of NLRP3 inhibitors in other indications, while providing important proof-of-concept for target validation, does not constitute direct evidence for the efficacy of NLRP3-targeted therapy in knee OA. Translating findings from other indications to knee OA requires careful consideration of disease-specific pathophysiological differences and must be validated through dedicated knee OA clinical trials.

At the level of targeted intervention strategies, the development of NLRP3 inhibitors has evolved from early probes to multiple clinical candidate drugs; however, no agent has yet entered a knee OA-specific clinical trial. Some next-generation inhibitors have shown preliminary safety and target engagement signals in early-stage clinical studies, but for knee OA as a localized degenerative disease, insufficient cartilage target exposure following systemic administration and potential immunosuppressive risks remain major translational bottlenecks. Although direct intra-articular injection can transiently increase local drug concentrations, it is limited by rapid lymphatic clearance from the joint cavity and the penetration barrier of the dense cartilage matrix, making it difficult to achieve sustained steady-state inhibition. In contrast, mesenchymal stem cell-derived EVs have shown potential in preclinical studies, which may offer a novel approach for local intra-articular delivery; however, scalable manufacturing, quality control, in vivo fate, and long-term safety remain to be addressed.

At the diagnostic translation dimension, the phospholipid bilayer of EVs effectively protects encapsulated pathway molecules, including NLRP3, active caspase-1, and mature IL-1β, conferring substantially greater stability and detection specificity in body fluids compared to free molecules. Clinical studies suggest that the EV-associated molecular profiles of vesicles in synovial fluid and circulation are correlated with Kellgren-Lawrence grade, synovial inflammation severity, and pain scores in OA, and have the potential to serve as candidate biomarkers for disease stratification and efficacy monitoring; however, detection methods have not yet been standardized, independent external validation is lacking, and their diagnostic performance and clinical utility still require validation in prospective cohorts.

It should be recognized that clinical implementation of NLRP3-targeted therapy for OA still faces multiple core challenges that urgently need to be addressed. First, knee OA exhibits high etiological heterogeneity and phenotypic diversity; NLRP3 hyperactivation may primarily be involved in the progression of inflammation-dominant subtypes rather than being a common pathological feature of all OA patients, and the lack of a standardized pathway activation stratification system is a key factor constraining the clinical efficacy of targeted drugs. Second, as an important danger sensor of the innate immune system, the physiological functions of NLRP3 play an important role in local joint infection defense and tissue damage repair; the immune safety margins of long-term local inhibition and the potential impact on chondrocyte physiological metabolism still lack large-sample long-term follow-up data. Third, the absence of large-scale manufacturing processes, batch consistency quality control standards, and in vivo tracing and safety evaluation systems for EV-based therapies also limits their translation from laboratory to clinical application.

Looking forward, future research in the field of NLRP3 targeting for OA can focus on the following directions: First, establishing NLRP3 inflammatory subtype classification standards based on EV multi-omics features, combined with radiomics and artificial intelligence algorithms, is a proposed research framework that may eventually enable non-invasive patient stratification and provide decision-making support for individualized targeted therapy; however, this approach remains unvalidated and requires prospective cohort studies, independent validation, standardized assays, and clinically meaningful endpoints. Second, developing engineered EV delivery systems that combine deep cartilage penetration, inflammation-responsive drug release, and prolonged joint retention can simultaneously enhance target inhibition efficiency and systemic safety. Third, exploring multi-mechanism combination therapeutic strategies that integrate NLRP3 inhibitors with cartilage repair agents and metabolic regulators can achieve bidirectional intervention by inhibiting inflammatory damage and promoting tissue repair, providing new strategies for potential disease-modifying therapy in knee OA; however, this remains investigational and has not been established in knee OA. In summary, with continued advances in structural biology, nanodelivery technologies, and companion diagnostic systems, precision targeting of the NLRP3 inflammasome may provide a potential direction for future research into disease-modifying strategies for knee OA. Nevertheless, no clinical evidence currently demonstrates that selective NLRP3 inhibition improves pain, function, structural progression, or other patient-centered outcomes in knee OA, and all disease-modification claims remain investigational.

## Figures and Tables

**Table 1:** Key molecular regulatory mechanisms of NLRP3 inflammasome activation.

Regulatory Level	Core Events/Molecules	Functions and Mechanisms	Corresponding Pathological Effects in Knee OA	Evidence Source	References
Structural basis	NLRP3 domains (PYD, NACHT, LRR)	PYD recruits ASC; NACHT contains ATPase activity mediating oligomerization; LRR senses signals and maintains autoinhibition	NLRP3 expression in chondrocytes and synovial macrophages; elevated NLRP3, caspase-1, and IL-1β in OA synovial tissue (human observational/animal studies)	Human observational and preclinical (OA animal/in vitro)	[33,34,35,36,37,38]
	NEK7 as a licensing factor	Embeds at LRR–NACHT interface, disrupts inactive conformation, promotes active disc-shaped oligomer formation	NEK7-NLRP3 interaction has been reported to participate in inflammatory regulation in OA synovial macrophages; direct structural evidence in chondrocytes remains limited (animal/in vitro)	Preclinical (OA animal/in vitro)	[35,88]
	ASC speck nanostructure	Dense core and radial filaments, prion-like polymerization, can be released extracellularly to propagate inflammation	EVs in OA synovial fluid can carry ASC; ASC speck release may promote inflammatory propagation within the joint cavity (in vitro/animal)	Preclinical (in vitro/animal); inferred in human OA	[30,89,90]
	NLRP3 phase separation	Activation signal induces liquid-like condensates acting as nucleation platforms for ASC recruitment	Direct evidence in OA is still lacking; this is inferred from other cell models	Preclinical (in vitro models of other diseases)	[30,31,91]
Priming signals	NF-κB pathway activation	Upregulates transcription of NLRP3, pro-IL-1β, pro-IL-18, increasing protein abundance	Mechanical stress, cartilage matrix degradation fragments, and DAMPs can activate NLRP3 transcription in chondrocytes and synovial macrophages via the TLR4/NF-κB pathway (animal/in vitro)	Preclinical (OA animal/in vitro)	[48,49,51,52]
	Post-translational modifications (ubiquitination, phosphorylation, SUMOylation, acetylation)	BRCC3 deubiquitination (dependent on NEK7) promotes activation; PKA/AKT phosphorylation at Ser5 inhibits; SENP6 de-SUMOylation promotes assembly	In OA, PKA/AKT-mediated inhibitory phosphorylation of NLRP3 and SENP6-mediated de-SUMOylation have been preliminarily studied in chondrocytes or synovial cells, but their specific roles still require validation (in vitro/animal)	Preliminary OA in vitro/animal; mechanisms mainly from other disease models	[54,56,57,58,61,92,93]
Activation signals	Potassium efflux	Most agonists depend on intracellular K^+^ decrease; high extracellular K^+^ blocks activation	In knee OA, mechanical stress, ATP-P2X7 signaling, and calcium signaling disturbances may trigger potassium efflux, and elevated synovial ATP levels may participate in this process (animal/in vitro)	Preclinical (OA animal/in vitro)	[60]
	Golgi membrane remodeling (dTGN)	Dispersed TGN carrying PtdIns4P; NLRP3 binds via polybasic region, enabling recruitment and initial oligomerization	Preliminary evidence exists for Golgi stress and dTGN formation in chondrocytes; however, direct evidence in synovial macrophages remains limited (in vitro/animal)	Preliminary OA in vitro/animal; mechanism mainly from other disease models	[66]
	Lysosomal membrane permeabilization	Particulate stimuli (uric acid crystals, silica) release cathepsin B, cleaving NLRP3 or its regulators	In knee OA, cartilage matrix degradation fragments, calcium pyrophosphate crystals, and uric acid crystals can trigger lysosomal damage, with cathepsin B release contributing to NLRP3 activation in chondrocytes and synovial macrophages (animal/in vitro)	Preclinical (OA animal/in vitro)	[62,63,67]
	Mitochondrial damage (mtROS, ox-mtDNA, MAVS)	MAVS scaffolds promote mitochondrial localization; ox-mtDNA directly binds NLRP3; TXNIP pathway mediates oxidative stress synergy	In knee OA, mechanical overload and metabolic disturbance can lead to the release of mitochondrial ROS and ox-mtDNA, contributing to NLRP3 activation in chondrocytes and synovial macrophages (animal/in vitro)	Preclinical (OA animal/in vitro)	[68,69,94,95,96,97,98,99]
Non-canonical pathway	Intracellular LPS activates caspase-4/5/11	Cleaves GSDMD to form membrane pores, causing K^+^ efflux and indirect NLRP3 activation; involved in sepsis cytokine storm	It may be involved in infectious arthritis; however, direct evidence in knee OA remains limited	Preclinical (in vitro/animal models of other diseases); inferred in OA	[47,100,101,102,103]
Downstream effects	Caspase-1 activation	Cleaves pro-IL-1β/pro-IL-18 to mature forms; cleaves GSDMD to release N-terminus, forming membrane pores	In knee OA, caspase-1 activation is associated with chondrocyte pyroptosis and IL-1β release from synovial macrophages (animal/in vitro)	Preclinical (OA animal/in vitro)	[73,74,75,76,77]
	Pyroptosis and sub-lethal pyroptosis	Lethal pyroptosis or ESCRT-mediated membrane repair allowing survival with release of factors, driving chronic low-grade inflammation	The non-lethal activation mode is an important basis for the persistent pathological role of NLRP3 in knee OA; chondrocyte pyroptosis contributes to cartilage loss (animal/in vitro)	Preclinical (OA animal/in vitro)	[78,79,80,81]
	ASC speck/vesicle-mediated intercellular propagation	Activated cells release ASC aggregates, active caspase-1, etc.; phagocytosed by neighboring cells inducing secondary activation	Intra-articular EVs can carry NLRP3, active caspase-1, and IL-1β, potentially promoting progression from focal injury to pan-articular degeneration; direct functional validation in humans remains lacking (in vitro/animal)	Preclinical (in vitro/animal); inferred in OA	[104,105]

**Table 2:** Clinical-stage direct NLRP3 inhibitors in non-OA indications.

Inhibitor	Developer	Primary Indications (Non-OA)	Clinical Phase (Non-OA)	Clinical Trial Registration Number (NCT)	Core Differentiating Advantages	Highest Evidence in Knee OA	References
ISM8969	Insilico Medicine/Hygtia Therapeutics	Parkinson’s disease, neuroinflammation	Phase I	NCT07581431	AI-designed, CNS-targeted	None	[123]
BGE-102	BioAge Labs	Cardiovascular risk, metabolic disease, obesity	Phase I	NCT07656727 (QUELL-CV), NCT07812090 (QUELL-DME)	Dual peripheral and CNS targeting	None	[124,125]
VTX3232	Ventyx Biosciences	Cardiovascular disease, obesity, Parkinson’s disease	Phase II a	NCT06556173, NCT06771115	Dual cardiovascular and neurological domains	None	[126,127]
NT-0796	NodThera	Obesity, cardiovascular disease	Phase II (RESOLVE-1); Phase II a (RESOLVE-2, with GLP-1RA)	NCT07055516 (RESOLVE-1), NCT07220629 (RESOLVE-2), NCT06129409	GLP-1RA combination strategy	None	[128,129,130]
Dapansutrile	Olatec Therapeutics	Gout, type 2 diabetes mellitus, Parkinson’s disease	Phase II/III (acute gout flare); Phase II (type 2 diabetes, Parkinson’s disease)	NCT05658575, NCT06047262 (DAPAN-DIA), NCT07157735 (DAPA-PD)	First NLRP3 inhibitor to enter clinical trials	None	[131,132,133]

**Table 3:** Preclinical NLRP3 inhibitors and indirect modulators investigated in knee OA.

Inhibitor Name	Direct/Indirect	Mechanism of Action/Binding Site	Chemical Class	Evidence in OA	Highest Evidence in Knee OA	Core Advantages/ Key Issues	References
MCC950 (CRID3)	Direct	Allosteric inhibition, targeting NACHT domain, stabilizing inactive conformation	Small molecule (sulfonylurea)	In OA animal models such as DMM and ACLT, intra-articular injection can attenuate cartilage degeneration, synovial inflammation, and osteophyte formation	Preclinical (OA animal models)	“Gold standard” probe; discontinued due to hepatotoxicity, off-target effects	[111,112,113,114,115,116]
Oridonin	Direct	Covalent modification of Cys279, blocking NLRP3–NEK7 interaction	Natural diterpenoid	No evidence in OA models	None	Representative natural covalent inhibitor	[20,118,134]
Dehydrocostus lactone	Direct	Covalent binding, selective blockade at extremely low concentration (10 nM)	Natural sesquiterpene lactone	No evidence in OA models	None	Highly potent covalent inhibitor	[119]
Grandiflorenic acid derivative (Compound 5)	Direct	Covalent binding at ATP-binding site, IC_50_ = 1.36 nM	Semi-synthetic derivative	No evidence in OA models	None	Ultra-high potency covalent inhibitor	[120]
Baicalein	Indirect	Upstream NF-κB/oxidative stress	Natural flavonoid	Intra-articular baicalein reduces OARSI score, IL-1β, TNF-α, MMP-13, ADAMTS-5 in DMM rats	Preclinical OA animal models	Not a selective NLRP3 inhibitor	[135]
Celastrol	Indirect	SIRT2-NLRP3 axis, reduces NLRP3 acetylation	Natural triterpenoid	Inhibits chondrocyte inflammation and GSDMD-mediated pyroptosis; increases IL-4 and IL-10 in rat OA	Preclinical OA animal models	Not a selective NLRP3 inhibitor	[136]
Glibenclamide	Indirect	MAPK pathway, NEK7-NLRP3 interaction	Small molecule (sulfonyl urea)	Reduces synovial macrophage infiltration, promotes M1 to M2, decreases IL-1β and TNF-α in OA animal models	Preclinical OA animal models	Not a selective NLRP3 inhibitor	[137,138]

**Table 4:** Pathological roles of the NLRP3 inflammasome in different joint tissues in OA.

Joint Tissue/Cell Type	NLRP3-Mediated Pathological Effects	Key Molecules/Pathways	Impact on OA Progression	References
Chondrocytes	Pyroptosis; upregulation of matrix-degrading enzymes	Caspase-1 activates GSDMD to form membrane pores; IL-1β and IL-18 induce the upregulation of MMP-3/9/13 and ADAMTS-5	Type II collagen and aggrecan degradation, progressive cartilage loss	[140,141,142,143,144,145,146,162]
Synovial macrophages	Sustained activation releasing large amounts of IL-1β and TNF-α; M1/M2 polarization imbalance	NLRP3/caspase-1 axis; MAPK pathway; NEK7–NLRP3 interaction	Synovial hyperplasia, chronic inflammation; paracrine damage to neighboring chondrocytes	[137,147,148,149,150,151,152,153,163,164]
Synovial fibroblasts	Persistent NLRP3 activation driven by autoantibody complexes and sustained damage signals	Not detailed in specific pathways (RA mechanism noted, similar in OA synovial fibroblasts)	Synovial inflammation and hyperplasia	[165,166]
Subchondral bone cells	Excessive osteoclast activation; osteoblast–osteoclast imbalance	NLRP3-mediated bone remodeling abnormalities	Subchondral bone sclerosis, osteophyte formation	[157,167,168]
EV-mediated trans-tissue propagation	Damaged chondrocytes/activated synovial cells release EVs carrying NLRP3, active caspase-1, IL-1β, etc.	EVs spread within joint cavity, taken up by healthy cells, inducing secondary inflammatory activation	Focal lesions lead to pan-articular degeneration, resulting in progressive worsening	[169,170,171,172]
Mechanical stress; metabolic signals; senescence signals	Piezo1-NF-κB/NLRP3 axis; AMPK/NLRP3 axis; lysosomal dysfunction-SASP	Mechanical overload, metabolic disturbances, senescent cell SASP	Multi-factor convergence on NLRP3, driving chronic low-grade inflammation	[9,161]

**Table 5:** Bidirectional functions of EVs in NLRP3-related diseases: exploratory diagnostic biomarkers and investigational therapeutic vectors.

Category	EV Source/Type	Cargo Molecules	Reported Association with NLRP3 Pathway	Application Direction	References
Pathological EVs (pro-inflammatory)	Synovial macrophages; damaged chondrocytes	Surface HMGB1, HSP70; carrying ATP, chondroitin sulfate fragments, miR-133a-3p, etc.	TLR4 binding activates NF-κB to upregulate NLRP3; P2X7 triggers K^+^ efflux; lysosomal rupture causes cathepsin B release.	Inflammatory signal propagation; disease progression biomarkers	[193,194,195,196,197,198,199,200,201,202,203]
	OA patient synovial fluid/serum EVs	NLRP3, active caspase-1, mature IL-1β, miR-133a-3p	Induce pyroptosis and inflammation in recipient cells (e.g., via miR-133a-3p degrading SIRT1 to activate NF-κB)	Disease diagnosis and staging	[172,221,236]
EVs investigated as therapeutic vectors (anti-inflammatory)	MSC-derived	miR-146a, miR-223, miR-1208, SIRT1, etc.	miR-146a targets IRAK1/TRAF6 to inhibit NF-κB; miR-223 binds NLRP3 mRNA 3′-UTR and inhibits translation; SIRT1 deacetylates NLRP3 and inhibits oligomerization.	Suppression of NLRP3 activation; chondroprotection; immunomodulation	[195,204,205,206,207,208,209,210,211,237]
	Metformin-pretreated synovial MSC-EVs	Enriched miR-1208	miR-1208 inhibits METTL3, leading to reduced NLRP3 m6A modification, which subsequently suppresses inflammasome activation.	OA therapy	[237]
	Glucocorticoid-pretreated synovial MSC-EVs	miR-212-5p, etc.	Downregulates NLRP3 inflammasome activity in macrophages	Inhibits cartilage degeneration and osteophyte formation	[203]
Engineered EVs investigated as therapeutic vectors	NLRP3 siRNA-loaded MSC-EVs	Electroporation-loaded siRNA	Gene silencing of NLRP3, improved IL-1β secretion inhibition rate	Targeted gene therapy	[225]
	Surface-modified with targeting peptides	Peptide modification for cartilage/synovial targeting	Enhances lesion enrichment	Local precision delivery	[226,227]
	ROS-responsive engineered EVs	Membrane-introduced disulfide bonds/boronate esters	Inflammatory microenvironment-responsive release of MCC950 and other inhibitors	Intelligent drug release, prolonged local action, reduced systemic toxicity	[117,228,229]
Biomarker applications	Synovial fluid EVs	NLRP3 protein, active caspase-1, IL-1β	Levels positively correlated with KL grade and synovial inflammation scores	Distinguishing early-vs. advanced-stage OA; reflecting WOMAC pain scores	[221,236,238]
	Peripheral blood circulating EVs	NLRP3 and type II collagen C-telopeptide	Co-expression levels for early OA diagnosis	Non-invasive early screening	[239,240,241]

## Data Availability

No new data were created or analyzed in this study. Data sharing is not applicable.

## Abbreviations

The following abbreviations are used in this manuscript:

3′-UTR	3′-untranslated region
AGEs	advanced glycation end products
AI	artificial intelligence
ASC	apoptosis-associated speck-like protein containing a CARD
ATP	adenosine triphosphate
AUC	area under the receiver operating characteristic curve
BBB	blood–brain barrier
CGRP	calcitonin gene-related peptide
CNS	central nervous system
cryo-EM	cryo-electron microscopy
DAMPs	damage-associated molecular patterns
DMM	destabilization of the medial meniscus
dTGN	dispersed trans-Golgi network
ECM	extracellular matrix
EVs	extracellular vesicles
FAP	fibroblast activation protein
GSDMD	gasdermin D
IPFP	infrapatellar fat pad
IRAK1	interleukin-1 receptor-associated kinase 1
Knee OA	Knee Osteoarthritis
LLPS	liquid–liquid phase separation
LPS	lipopolysaccharide
MAVS	mitochondrial antiviral signaling protein
mRNA	messenger RNA
MSCs	mesenchymal stem cells
mtROS	mitochondrial reactive oxygen species
NLRP3	NLR family pyrin domain-containing protein 3
NSAIDs	nonsteroidal anti-inflammatory drugs
OA	osteoarthritis
ox-mtDNA	oxidized mitochondrial DNA
PtdIns4P	phosphatidylinositol 4 phosphate
PTMs	post-translational modifications
ROS	reactive oxygen species
SASP	senescence-associated secretory phenotype
siRNA	small interfering RNA
SIRT1	sirtuin 1
sEVs	small extracellular vesicles
TLR	Toll-like receptor
TRAF6	TNF receptor-associated factor 6
XOR	xanthine oxidoreductase
